# Supplementary material for: Photo-triggered full-color circularly polarized luminescence based on photonic capsules for multilevel information encryption
Source: Nat Commun. 2023 May 25;14:3005. doi: 10.1038/s41467-023-38801-1 (PMC10212932; doi:10.1038/s41467-023-38801-1)
Supplement: Supplementary file 1 — Supplementary Information [file 41467_2023_38801_MOESM1_ESM.pdf]

Supplementary information

Photo-Triggered Full-Color Circularly Polarized Luminescence Based on Photonic Capsules for Multilevel Information Encryption

Siyang Lin[1], Yuqi Tang[2], Wenxin Kang[1], Hari Krishna Bisoyi[3], Jinbao Guo[1,*] and Quan Li[2,3,*]

[1]Key Laboratory of Carbon Fibers and Functional Polymers, Ministry of Education; College of Materials Science and Engineering, Beijing University of Chemical Technology, Beijing 100029, China. *E-mail: guojb@mail.buct.edu.cn

[2]Institute of Advanced Materials and School of Chemistry and Chemical Engineering, Southeast University, Nanjing 211189, China. *E-mail: quanli3273@gmail.com

[3]Advanced Materials and Liquid Crystal Institute and Materials Science Graduate Program, Kent State University, Kent, Ohio 44242, USA.

1. Molecular synthesis and structural characterization

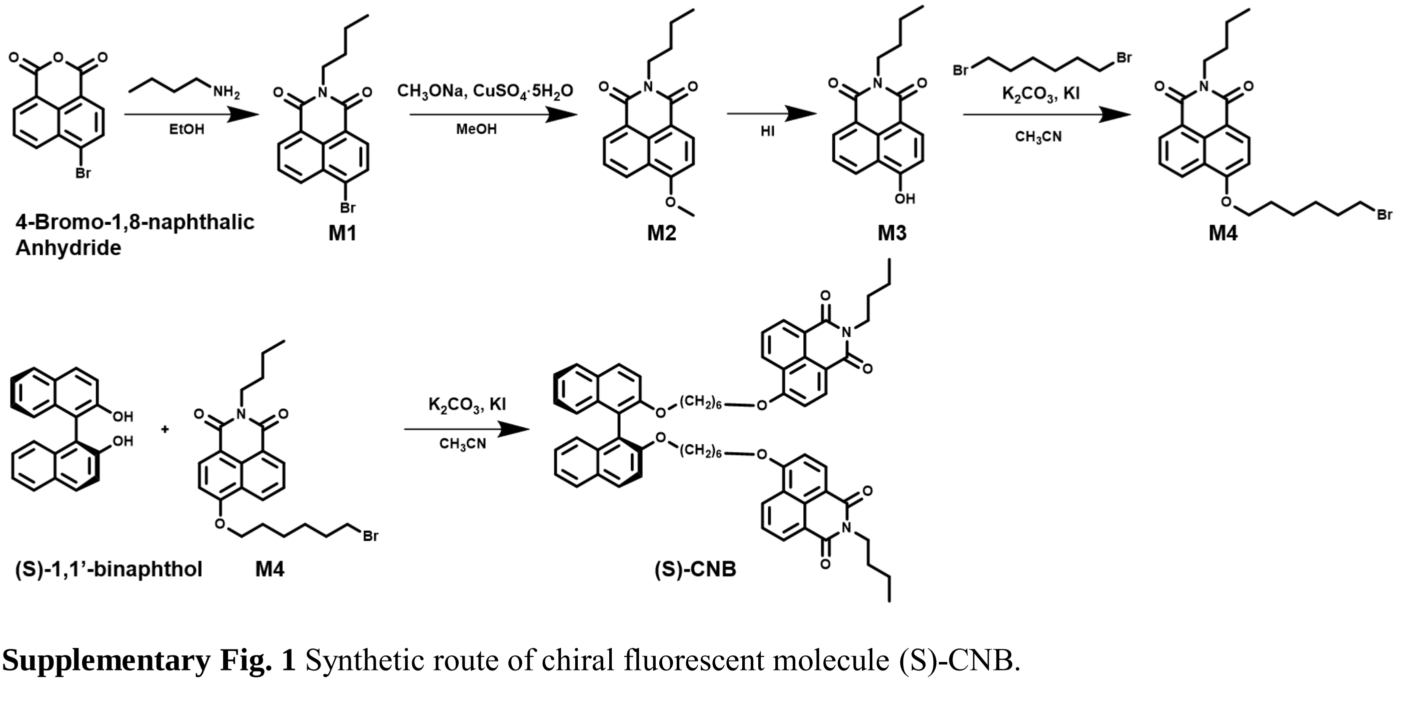

Supplementary Fig. 1 Synthetic route of chiral fluorescent molecule (S)-CNB.

The synthetic procedures of compound M1-M3 were synthesized according to the previous references.[1, 2]

M1: 4-Bromo-1,8-naphthalic anhydride (2.7 g, 10 mmol) and n-butylamine (1.46 g, 20 mmol) were dissolved in 100 mL dry ethanol and refluxed at 85 °C overnight. The mixture was extracted with dichloromethane and deionized water. After column chromatography (acetic ether: petroleum ether=8: 1), M1 (yellow solid, 2.48 g, 74.9%) was obtained. ^{1}H-NMR (400 MHz, $CDCl_3$) δ 8.66 (d, 1H), 8.57 (d, 1H), 8.41 (d, 1H), 8.04 (d, 1H), 7.88 – 7.81 (m, 1H), 4.20 – 4.14 (m, 2H), 1.76 – 1.68 (m, 2H), 1.49 – 1.41 (m, 2H), 0.98 (t, 3H).

M2: M1 (2.48 g, 7.5 mmol) and $CuSO_4·5H_2O$ (0.19 g, 0.75 mmol) were dissolved in 30 mL methanol solution of sodium methoxide (0.5 M, 15mmol) and refluxed at 60 °C for 8 h under nitrogen atmosphere. After filtration, the resulting solid was dissolved in 30 mL HCl solution (1M) and stirred for 30 min. The mixture was filtered, and the obtained solid was dried by a vacuum oven and purified by column chromatography (dichloromethane) to gain the compound M2 (yellow solid, 0.52 g, 24.5%). ^{1}H-NMR (400 MHz, $CDCl_3$) δ 8.60 (dd, 1H), 8.55 (dd, 2H), 7.70 (dd, 1H), 7.04 (d, 1H), 4.21 – 4.14 (m, 2H), 4.13 (s, 3H), 1.71 (p, 2H), 1.45 (dq, 2H), 0.97 (t, 3H).

M3: M2 (0.52 g, 1.8 mmol) was soluble in 4.2 mL HI solution (57 wt%) and refluxed at 130 °C for 7 h. After cooled down to the room temperature, the mixture was poured into ice water and the precipitate was collected by filtration. After dried in a vacuum oven at 60 °C, the crude product M3 (yellow solid, 0.26 g, 54.2%) was obtained without further purification. ^{1}H-NMR (400 MHz, Acetone-d_6) δ 10.54 (s, 1H), 8.62 (dd, 1H), 8.55 (dd, 1H), 8.42 (d, 1H), 7.80 (dd, 1H), 7.23 (d, 1H), 4.15 – 4.09 (m, 2H), 1.69 (p, 2H), 1.43 (dt, 2H), 0.97 (d, 3H).

M4: M3 (0.26 g, 0.97 mmol), 1,6-dibromohexane (2.3 g, 9.7 mmol), and K_2CO_3 (0.28 g, 2 mmol) were dissolved in 15 mL acetonitrile, and 1 mL N,N-dimethylformamide was added into the solution. The mixture was refluxed at 80 °C overnight. After filtration to remove the solid, the filtrate was extracted with dichloromethane and deionized water. M4 (white solid, 0.34 g, 81.0%) was obtained by column chromatography (dichloromethane: petroleum ether=8: 1). ^{1}H-NMR (400 MHz, $CDCl_3$) δ 8.60 (d, *J* = 7.3 Hz, 1H), 8.55 (t, *J* = 9.1 Hz, 2H), 7.74 – 7.67 (m, 1H), 7.02 (d, *J* = 8.3 Hz, 1H), 4.28 (t, *J* = 6.3 Hz, 2H), 4.21 – 4.13 (m, 2H), 3.45 (t, *J* = 6.7 Hz, 2H), 1.97 (dq, *J* = 20.5, 6.8 Hz, 4H), 1.71 (p, *J* = 7.6 Hz, 2H), 1.61 (dd, *J* = 7.4, 4.1 Hz, 4H), 1.50 – 1.40 (m, 2H), 0.97 (t, *J* = 7.3 Hz, 3H). ^{13}C NMR (101 MHz, $CDCl_3$) δ 164.54 , 163.96 , 160.16 , 133.42 , 131.47 , 129.41 , 128.56 , 125.85 , 123.54 , 122.48 , 114.96 , 105.79 , 68.79 , 40.09 , 33.69 , 32.61 , 30.29 , 28.87 , 27.92 , 25.42 , 20.43 , 13.89. HR-MS (ESI, positive mode): calculated for $C_{22}H_{26}BrNO_3$ $[M+H]^+$ 432.1169; found 432.1179.

(S)-CNB: M4 (0.34 g, 0.79 mmol), (S)-1,1'-binaphthol (0.1 g, 0.36 mmol), and K_2CO_3 (0.15 g, 1.08 mmol) were dissolved in 10 mL acetonitrile. After being refluxed at 80 °C overnight, the mixture was filtered out the solid and concentrated. The chiral fluorescent molecule (S)-CNB (light-yellow solid, 0.183 g, 51.4%) was gained by column chromatography (ethyl acetate: petroleum ether=1: 4). ^{1}H-NMR (400 MHz, $CDCl_3$) δ 8.59 (d, *J* = 8.4 Hz, 2H), 8.53 (d, *J* = 8.3 Hz, 2H), 8.49 (d, *J* = 8.4 Hz, 2H), 7.88 (d, *J* = 9.0 Hz, 2H), 7.79 (d, *J* = 8.1 Hz, 2H), 7.70 – 7.63 (m, 2H), 7.39 (d, *J* = 9.0 Hz, 2H), 7.28 – 7.25 (m, 2H), 7.22 – 7.13 (m, 4H), 6.93 (d, *J* = 8.3 Hz, 2H), 4.23 – 4.14 (m, 4H), 4.06 – 3.95 (m, 6H), 3.94 – 3.86 (m, 2H), 1.72 (p, *J* = 7.6 Hz, 4H), 1.60 (s, 4H), 1.51 – 1.40 (m, 8H), 1.23 – 1.14 (m, 4H), 0.98 (t, *J* = 7.3 Hz, 10H). ^{13}C-NMR (151 MHz, $CDCl_3$) δ 164.56, 164.01 , 160.25 , 154.52 , 134.23 , 133.43 , 131.48 , 129.43 , 129.34 , 129.10 , 128.58 , 127.78 , 126.14 , 125.80 , 125.56 , 123.56 , 122.52 , 120.86 , 115.92 , 114.86 , 105.80 , 69.70 , 68.72 , 40.11 , 30.31 ,

29.27 , 28.65 , 26.93 , 25.39 , 25.38 , 20.44 , 13.89. HR-MS (ESI, positive mode): calculated for $C_{64}H_{64}N_2O_8$ $[M+H]^+$ 989.4735; found 989.4732.

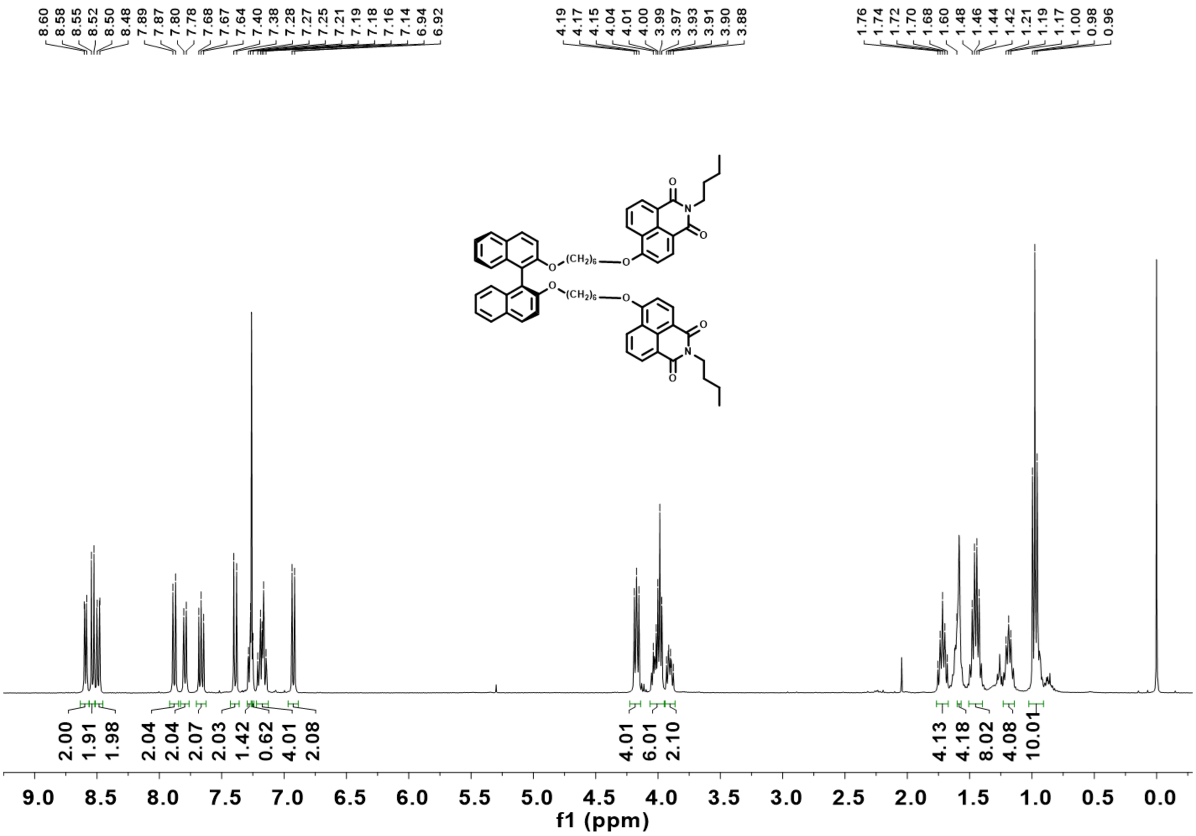

Supplementary Fig. 2 ^{1}H-NMR of (S)-CNB (400 MHz, $CDCl_3$).

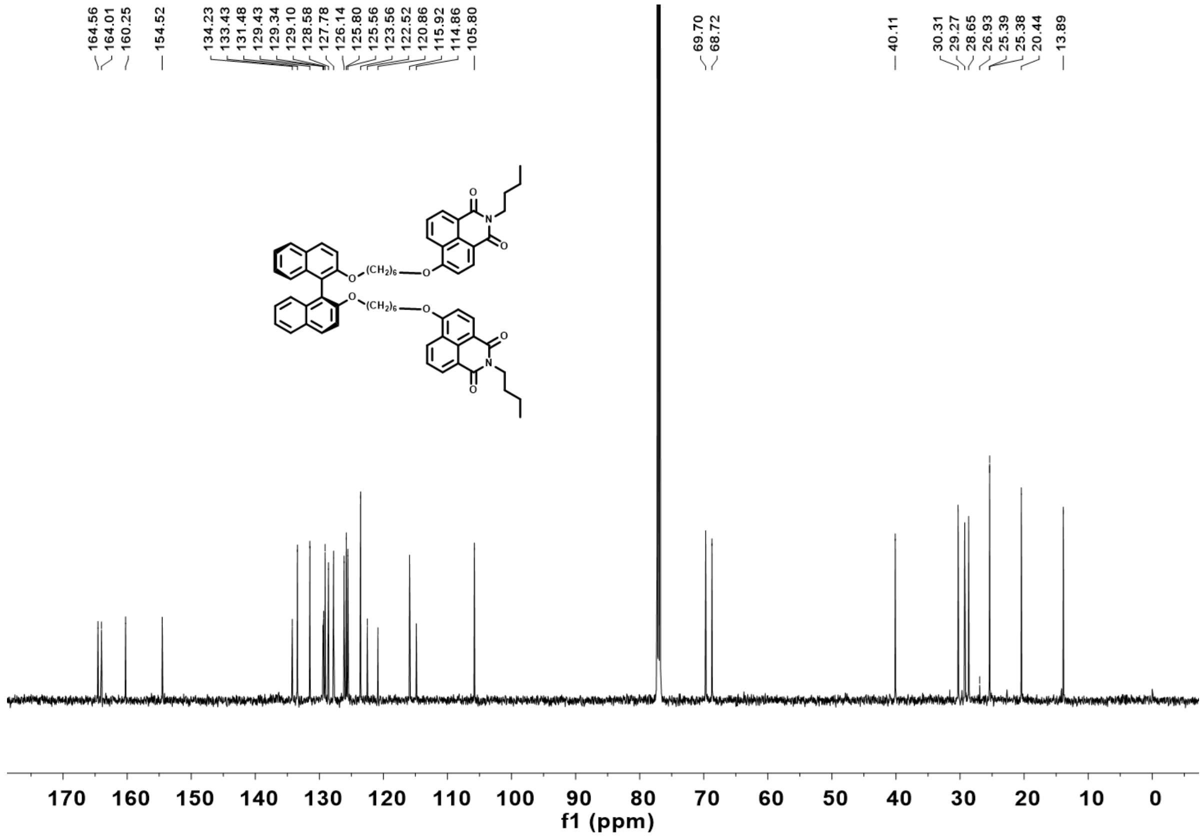

Supplementary Fig. 3 ^{13}C-NMR of (S)-CNB (151 MHz, $CDCl_3$).

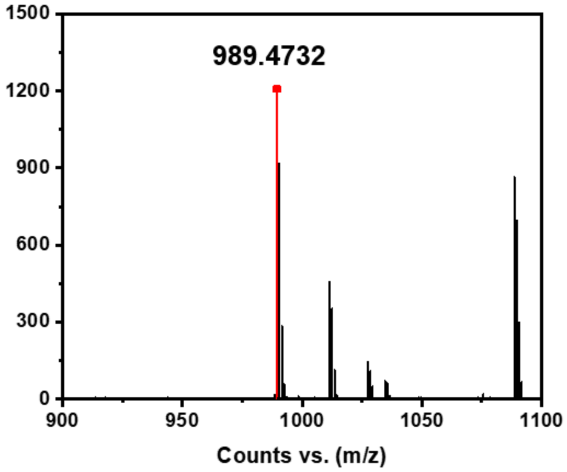

Supplementary Fig. 4 HR-MS of (S)-CNB in CH_3CN: Calculated for $C_{64}H_{64}N_2O_8$ $[M+H]^+$ 989.4735; found 989.4732.

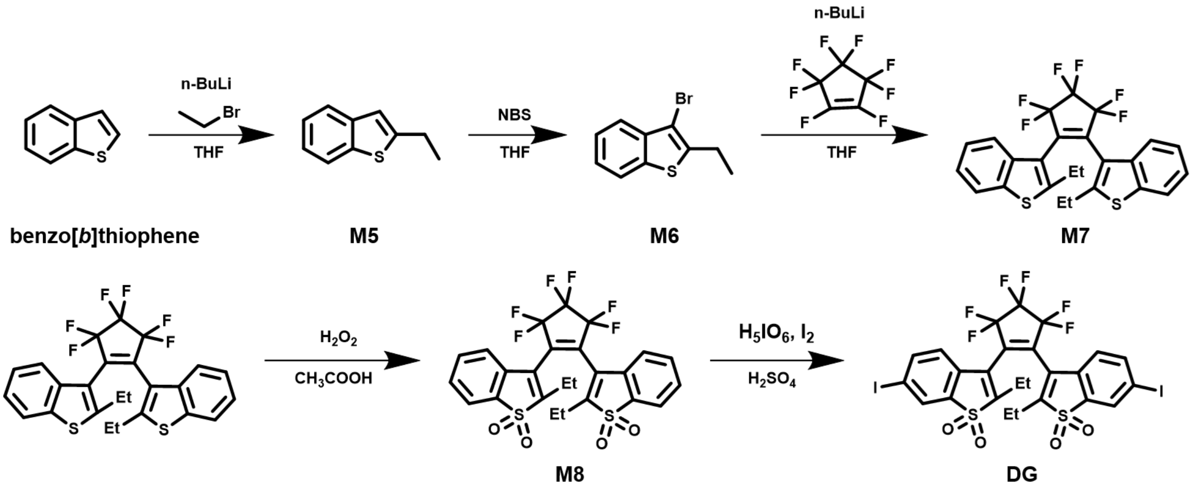

Supplementary Fig. 5 Synthetic route of fluorescent molecular switch DG.

DG was synthesized according to our previous work.[3]

M5: Benzo[*b*]thiophene (2.68 g, 20 mmol) was dissolved in 40 mL dry THF at -78 °C for 30 minutes under the protection of nitrogen and n-BuLi (10 mL, 24 mmol, 2.4 M,) was added in solution drop by drop. After stirring for 30 minutes, bromoethane (4.32 g, 40 mmol) was added into solution dropwise. The mixture was warmed up to room temperature slowly and reacted for 18 h, then HCl solution (30 mL, 0.2 M) was added. The mixture was extracted with chloroform and purified by column chromatography (petroleum ether) to obtain M5 (colorless liquid, 2.5 g, 77.2%). ^{1}H-NMR (400 MHz, $CDCl_3$) δ 7.89 (d, *J* = 8.3 Hz, 1H), 7.79 (d, *J* = 7.7 Hz, 1H), 7.47 – 7.36 (m, 2H), 7.11 (s, 1H), 3.08 – 3.02 (m, 2H), 1.51 (t, *J* = 7.5 Hz, 3H).

M6: M5 (2.5 g, 15.4 mmol) was dissolved in 10 mL dry THF under the protection of nitrogen and light avoidance and stirred at -5 °C for 30 minutes. NBS (3 g, 16.9 mmol) was soluble in 40 mL dry THF and was added to the solution drop by drop. The solution was warmed up to room temperature slowly and stirred for 15 h. After reaction, saturated $Na_2S_2O_3$ solution (30 mL) was added and the mixture was extracted with chloroform. M6 (colorless liquid, 3.09 g, 83.7%) was obtained by column chromatography (petroleum ether). ^{1}H-NMR (400 MHz, $CDCl_3$) δ 7.81 (dd, *J* = 8.1, 4.1 Hz, 2H), 7.47 (t, *J* = 7.2 Hz, 1H), 7.39 (t, *J* = 7.6 Hz, 1H),

3.04 (q, J = 7.6 Hz, 2H), 1.42 (t, J = 7.6 Hz, 3H).

M7: M6 (3.09 g, 12.8 mmol) was dissolved in 40 mL dry THF at -78 °C for 30 minutes under the protection of nitrogen and n-BuLi (6 mL, 14.1 mmol, 2.4 M,) was added in solution drop by drop. After stirring for 30 minutes, perfluorcyclopenten (1.35 g, 6.4 mmol) was added into solution dropwise. The mixture was warmed up to room temperature slowly and reacted for 16 h, then HCl solution (30 mL, 0.2 M) was added. The mixture was extracted with chloroform and purified by column chromatography (petroleum ether) to obtain M7 (white solid, 0.78 g, 24.6%). ^{1}H-NMR (400 MHz, $CDCl_3$) δ 7.76 – 7.54 (m, 4H), 7.36 (dq, J = 32.6, 8.1, 7.2 Hz, 3H), 7.19 (d, J = 9.0 Hz, 1H), 2.95 – 2.47 (m, 4H), 1.35 – 1.28 (m, 2H), 0.81 (t, J = 8.1 Hz, 4H).

M8: M7 (0.78 g, 1.6 mmol) was dissolved in 40 mL CH_3COOH and H_2O_2 solution (7.2 mL, 30%) was added dropwise at 118 °C. After refluxing for 30 min, the mixture was cooled down to the room temperature and poured into ice water (40 mL). The precipitate was collected by filtration and washed by deionized water. After dried in a vacuum oven at 60 °C, the crude product M8 (light-yellow solid, 0.63 g, 70.8%) was obtained without further purification. ^{1}H-NMR (400 MHz, $CDCl_3$) δ 7.75 (d, J = 7.2 Hz, 1H), 7.68 (d, J = 7.7 Hz, 1H), 7.64 – 7.37 (m, 4H), 7.20 (d, J = 7.4 Hz, 1H), 7.14 (d, J = 7.4 Hz, 1H), 2.59 (ddt, J = 30.1, 15.1, 7.5 Hz, 3H), 2.38 (dd, J = 15.0, 7.5 Hz, 1H), 1.43 – 1.37 (m, 2H), 1.05 (t, J = 7.6 Hz, 4H).

DG: M8 (0.63 g, 1.1 mmol) was dissolved in 12 mL H_2SO_4 and stirred at -5 °C for 30 min under the light avoidance. H_5IO_6 (0.31 g, 1.38 mmol) and I_2 (0.63 g, 2.48 mmol) were added to the solution and the mixture was reacted for 2 h. The mixture was poured into ice water (25 mL) and was extracted with chloroform, the aqueous phase was extracted with ethyl acetate again. Then the mixture was washed by $Na_2S_2O_3$, $NaHCO_3$, and NaCl solution to gain a colorless solution. The resulting residue was purified by column chromatography

(ethyl acetate: *n*-hexane=1:8) to give a final product DG (white solid, 0.38 g, 42.7%). ^{1}H-NMR (400 MHz, $CDCl_3$) δ 8.06 (s, 1H), 8.00 (s, 1H), 7.95 (d, J = 9.5 Hz, 1H), 7.78 (d, J = 6.6 Hz, 1H), 6.91 (d, J = 8.1 Hz, 1H), 6.81 (d, J = 8.0 Hz, 1H), 2.63 – 2.41 (m, 3H), 2.33 (dd, J = 15.1, 7.6 Hz, 1H), 1.38 (t, J = 7.6 Hz, 3H), 1.06 (t, J = 7.6 Hz, 3H). ^{13}C-NMR (151 MHz, $CDCl_3$) δ 148.46 , 147.98 , 142.78 , 142.44 , 140.01 , 139.99 , 139.81 , 139.63 , 136.91 , 131.34 , 131.31 , 128.79 , 128.66 , 123.89 , 123.49 , 122.85 , 122.69 , 96.45 , 96.41 , 19.25 , 19.09 , 11.71 , 11.48. ^{19}F-NMR (377 MHz, $CDCl_3$) δ -109.90 (d, 4F), -131.57 – -133.02 (m, 2F). HR-MS (ESI, positive mode): calculated for $C_{25}H_{16}F_6I_2O_4S_2$ $[M+Na]^+$ 834.8376; found 834.8169.

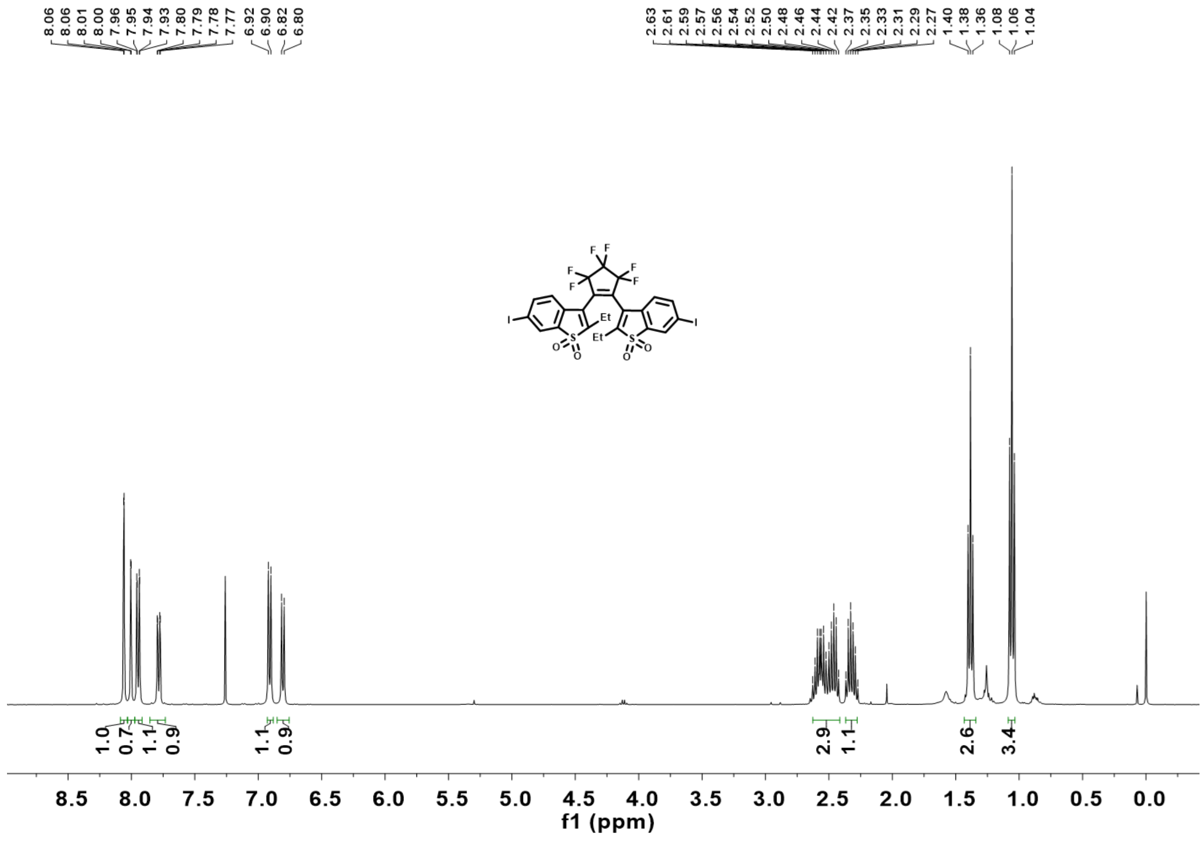

Supplementary Fig. 6 ^{1}H-NMR of DG (400 MHz, $CDCl_3$).

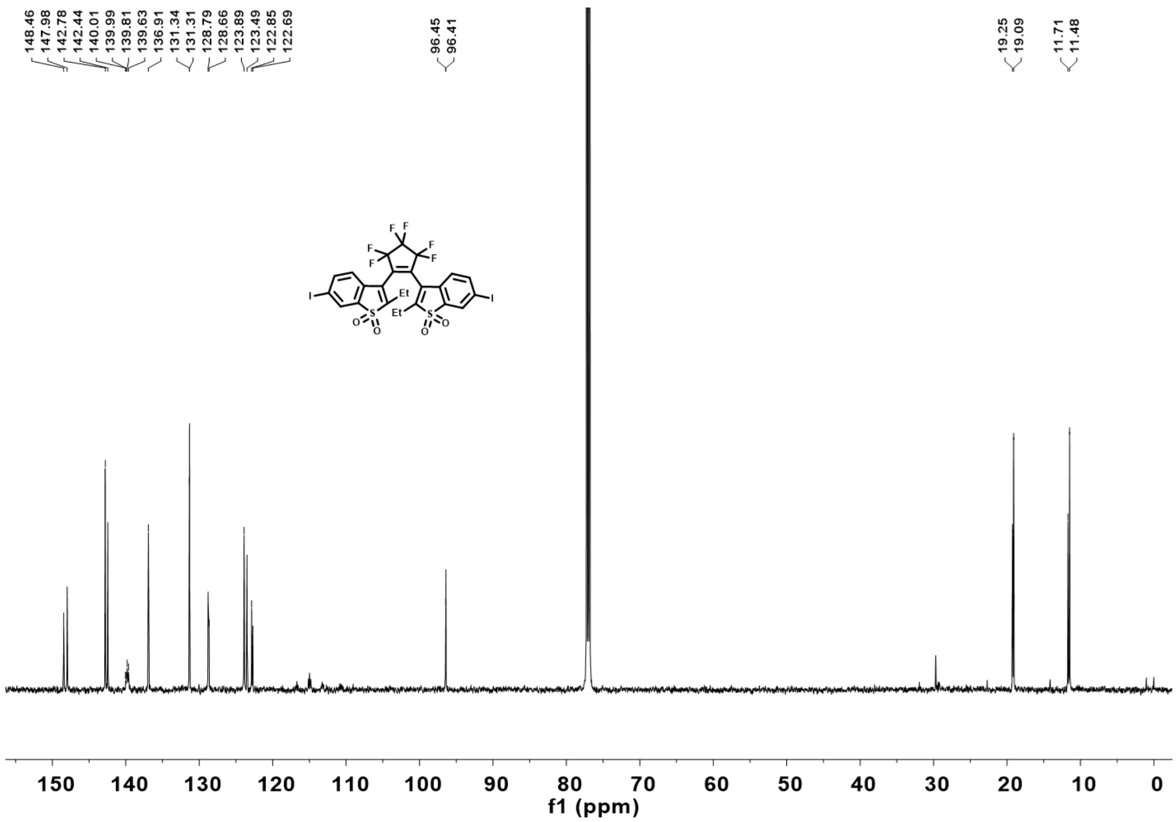

Supplementary Fig. 7 ^{13}C-NMR of DG (151 MHz, $CDCl_3$).

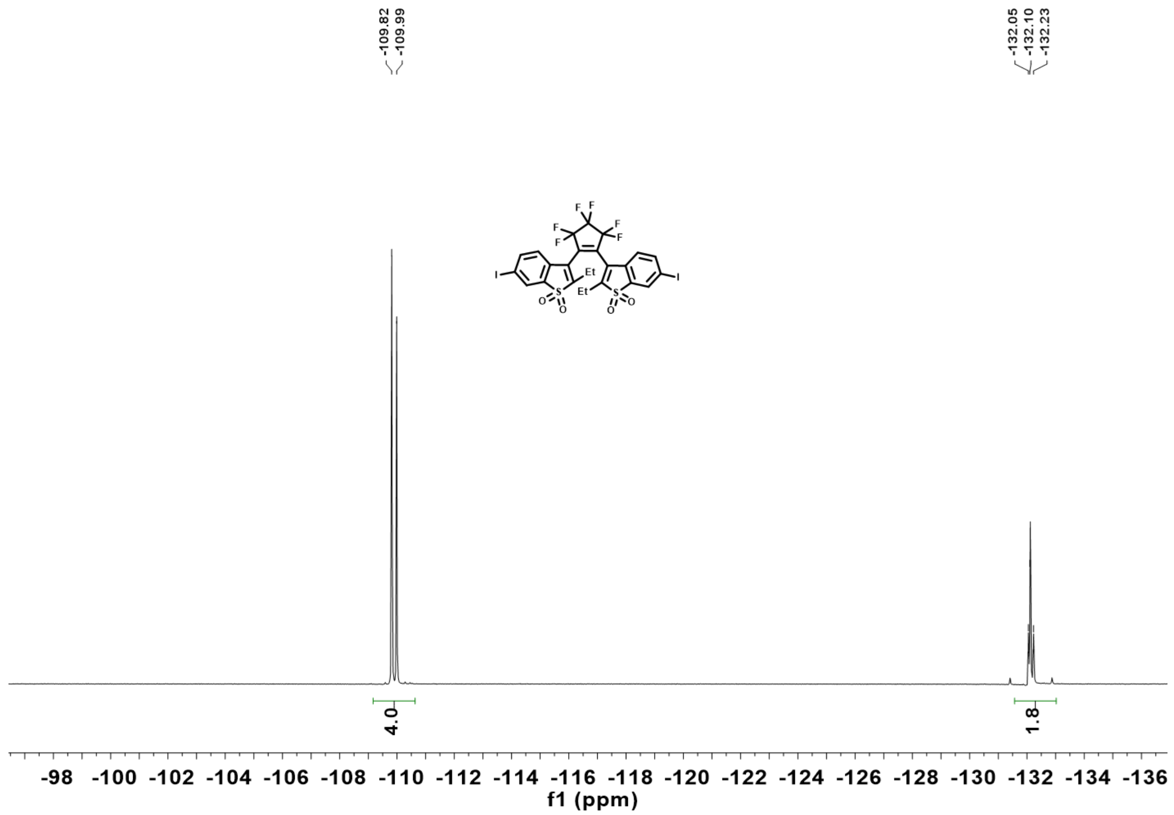

Supplementary Fig. 8 ^{19}F-NMR of DG (377 MHz, $CDCl_3$).

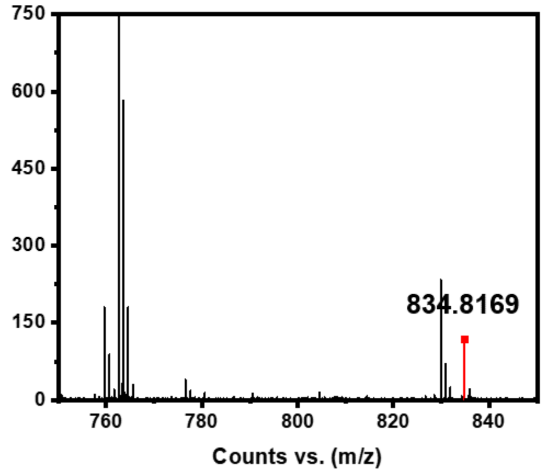

Supplementary Fig. 9 HR-MS of DG in CH_3CN: Calculated for $C_{25}H_{16}F_6I_2O_4S_2$ $[M+Na]^+$ 834.8376; found 834.8169.

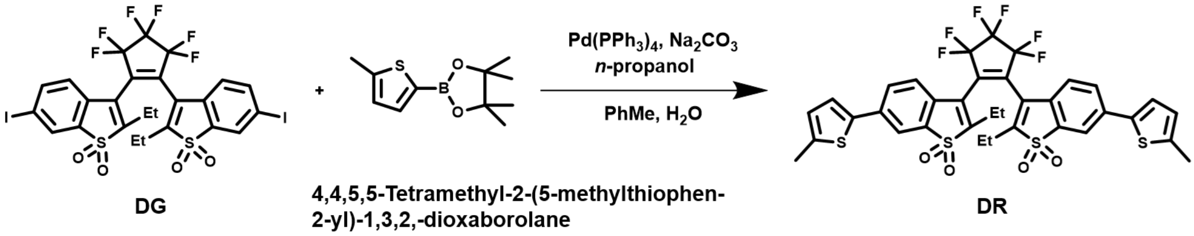

Supplementary Fig. 10 Synthetic route of fluorescent molecular switch DR.

DR: DG (1.91 g, 2.4 mmol), 4,4,5,5-tetramethyl-2-(5-methylthiophen-2-yl)-1,3,2,-dioxaborolane (1.61 g, 7.2 mmol) and tetrakis(triphenylphosphine)palladium(0) (0.14 g, 0.12 mmol) were dissolved in 100 mL toluene. The mixture was then added 20 mL *n*-propanol and 30 mL 20% Na_2CO_3 solution. After being stirred at 90 °C under nitrogen for 24 h, the mixture was extracted with chloroform and concentrated. The resulting residue was purified by column chromatography (ethyl acetate: *n*-hexane=1:5) to give a final product (red solid, 1.38 g, yield 76.7%). ^{1}H-NMR (400 MHz, $CDCl_3$) δ 7.90 (d, J = 1.7 Hz, 1H), 7.82 (d, J = 1.7 Hz, 1H), 7.71 (dd, J = 8.1, 1.8 Hz, 1H), 7.54 (dd, J = 8.1, 1.7 Hz, 1H), 7.23 (d, J = 3.6 Hz, 1H), 7.20 – 7.13 (m, 2H), 7.09 (d, J =

8.1 Hz, 1H), 6.76 (ddd, *J* = 18.6, 3.7, 1.2 Hz, 2H), 2.75 – 2.27 (m, 10H), 1.44 – 1.38 (m, 2H), 1.06 (t, *J* = 7.6 Hz, 4H). ^{13}C-NMR (101 MHz, $CDCl_3$) δ 147.81 , 147.49 , 142.67 , 142.53 , 138.42 , 137.83 , 137.77 , 136.62 , 136.55 , 129.69 , 129.42 , 127.04 , 126.94 , 125.47 , 125.41 , 123.41 , 123.34 , 123.22 , 122.91 , 118.96 , 118.90 , 19.24 , 19.05 , 15.60 , 15.56 , 11.96 , 11.68. ^{19}F-NMR (377 MHz, $CDCl_3$) δ -108.57 – -111.15 (m, 4F), -132.17 (d, 2F). HR-MS (ESI, positive mode): calculated for $C_{35}H_{26}F_6O_4S_4$ $[M+Na]^+$ 775.0510; found 775.0501.

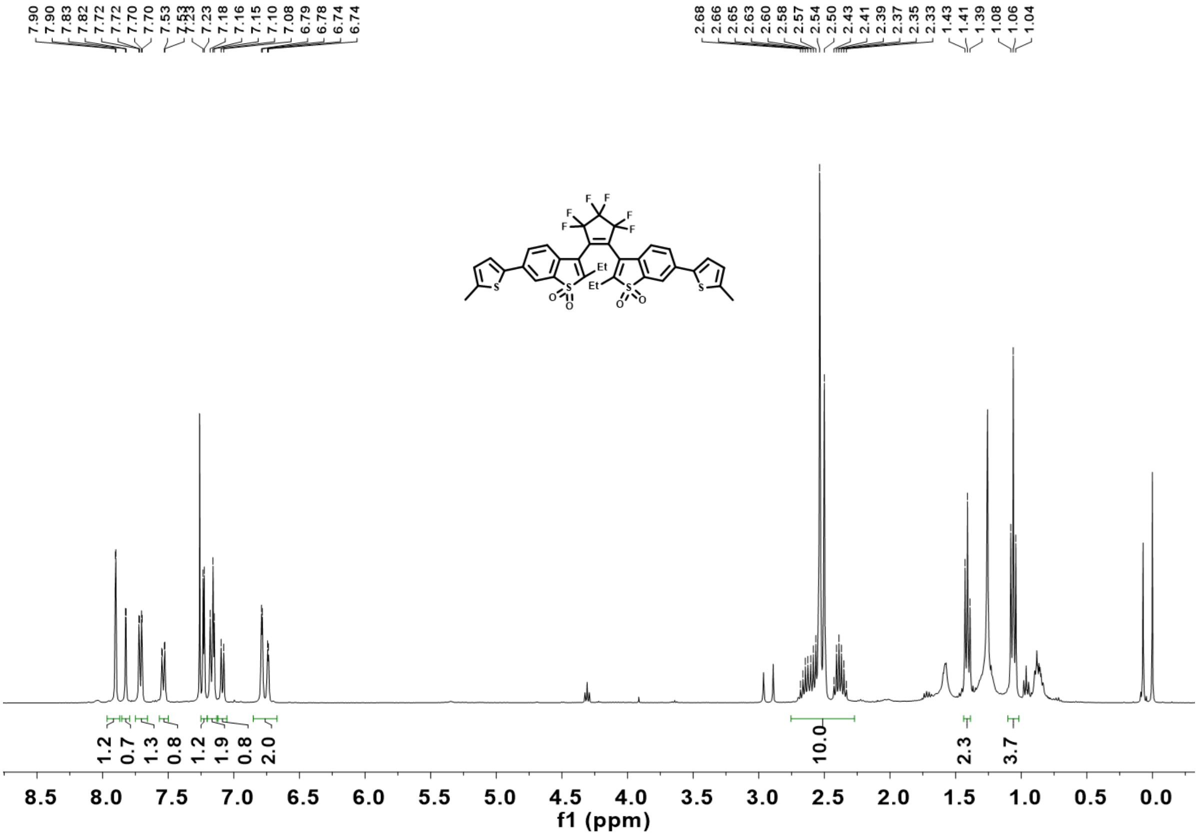

Supplementary Fig. 11 ^{1}H-NMR of DR (400 MHz, $CDCl_3$).

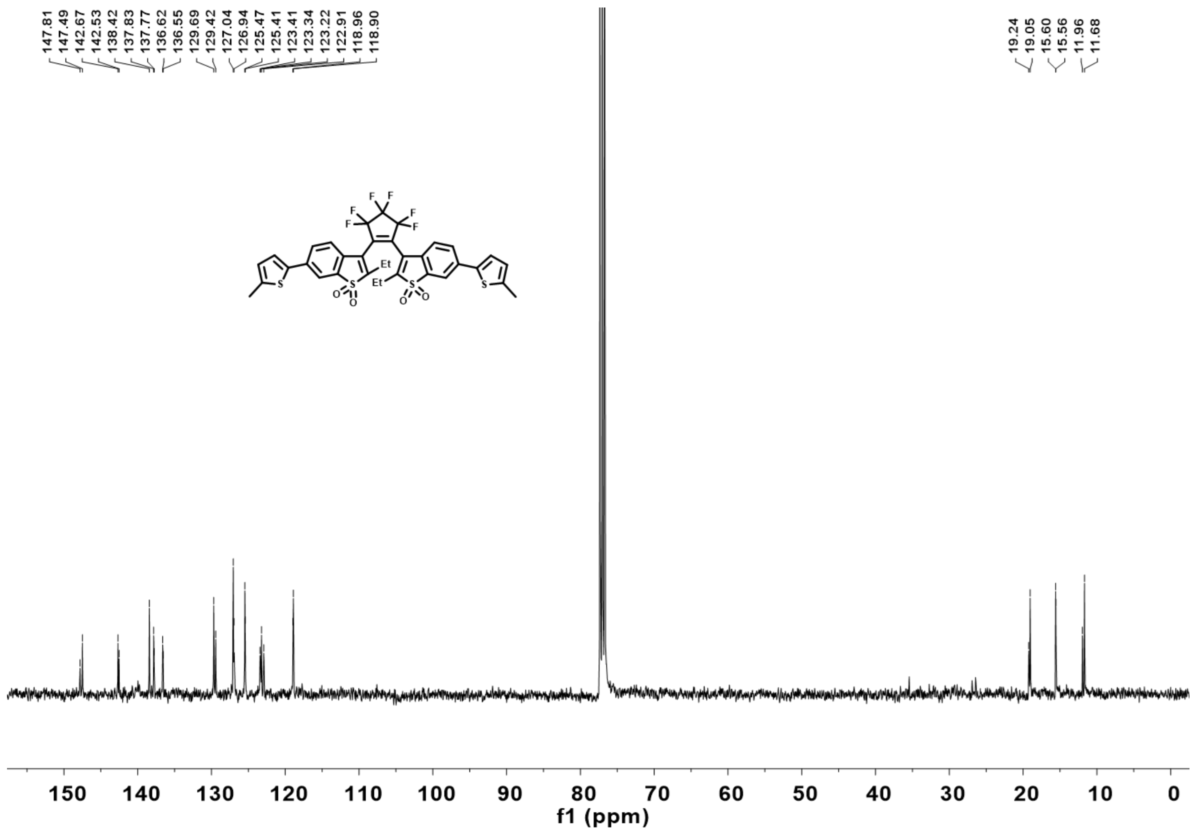

Supplementary Fig. 12 ^{13}C-NMR of DR (151 MHz, $CDCl_3$).

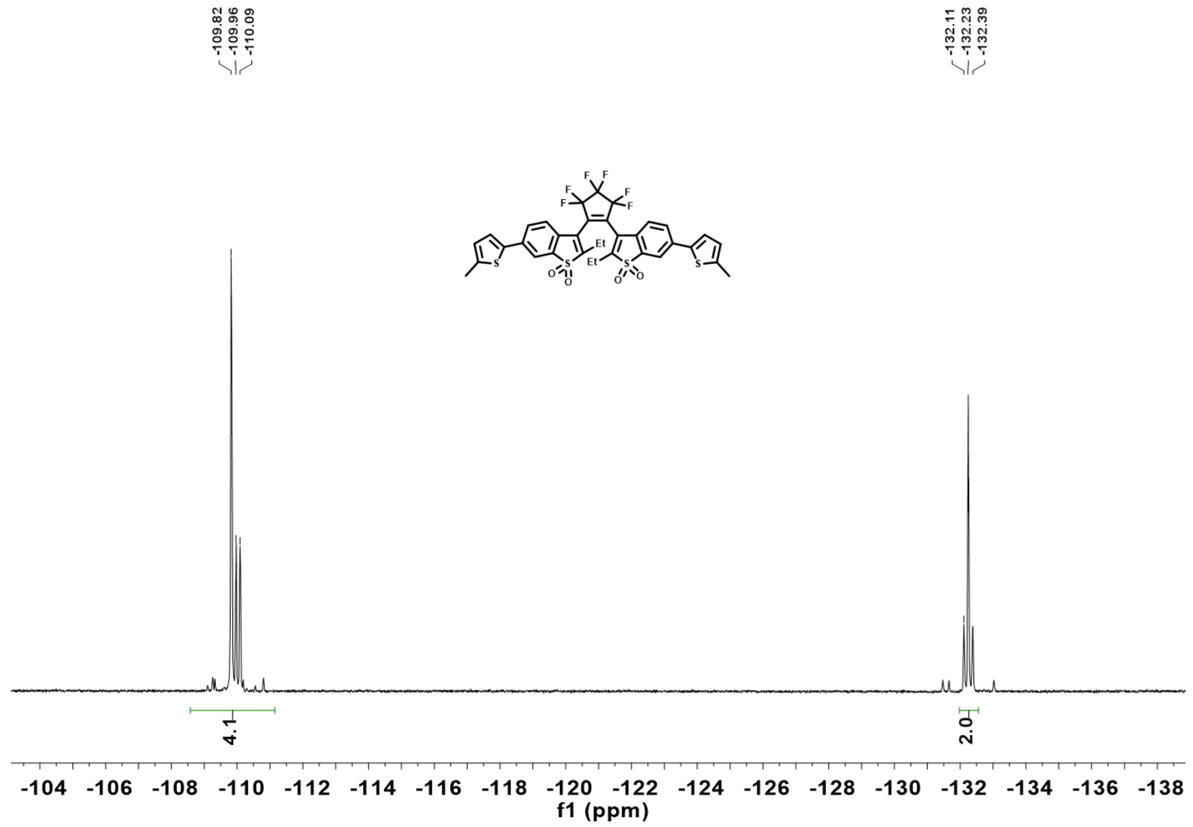

Supplementary Fig. 13 ^{19}F-NMR of DR (377 MHz, $CDCl_3$).

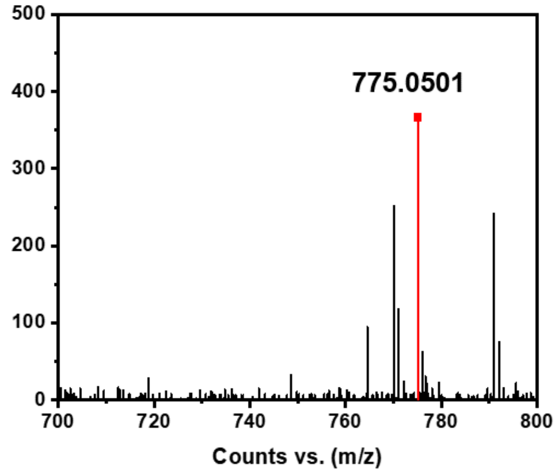

Supplementary Fig. 14 HR-MS of DR in CH_3CN: Calculated for $C_{35}H_{26}F_6O_4S_4$ $[M+Na]^+$ 775.0510; found 775.0501.

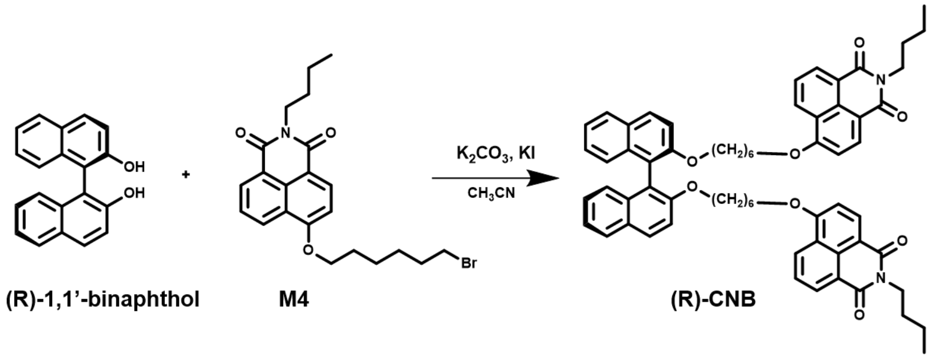

Supplementary Fig. 15 Synthetic route of chiral fluorescent molecule (R)-CNB.

(R)-CNB: M4 (0.5 g, 1.16 mmol), (S)-1,1'-binaphthol (0.14 g, 0.51 mmol), and K_2CO_3 (0.2 g, 1.5 mmol) were dissolved in 30 mL acetonitrile. After being refluxed at 80 °C overnight, the mixture was filtered out the solid and concentrated. The chiral fluorescent molecule (*R*)-CNB (light-yellow solid, 0.274 g, 54.4%) was gained by column chromatography (ethyl acetate: petroleum ether=1: 4). ^{1}H-NMR (400 MHz, $CDCl_3$) δ 8.59 (d, *J* = 6.2 Hz, 2H), 8.53 (d, *J* = 8.3 Hz, 2H), 8.49 (d, *J* = 8.4 Hz, 2H), 7.88 (d, *J* = 9.0 Hz, 2H), 7.80 (d, *J* = 8.1 Hz, 2H), 7.69 – 7.63 (m, 2H), 7.39 (d, *J* = 9.0 Hz, 2H), 7.28 (d, *J* = 8.0 Hz, 2H), 7.25 (s, 0H), 7.21 – 7.14 (m, 4H), 6.92 (d, *J* = 8.3 Hz, 2H), 4.21 – 4.13 (m, 4H), 4.07 – 3.95 (m, 6H), 3.93 – 3.86 (m, 2H), 1.71 (q, *J* = 7.7 Hz,

4H), 1.63 – 1.56 (m, 4H), 1.50 – 1.38 (m, 8H), 1.23 – 1.14 (m, 4H), 0.98 (t, *J* = 7.3 Hz, 10H). ^{13}C-NMR (101 MHz, $CDCl_3$) δ 164.54, 163.99, 160.23, 154.50, 134.21, 133.41, 131.46, 129.41, 129.32, 129.08, 128.57, 127.76, 126.12, 125.78, 125.54, 123.54, 122.50, 120.84, 115.90, 114.85, 105.79, 69.69, 68.71, 40.11, 30.30, 29.26, 29.12, 28.64, 28.53, 25.38, 20.43, 13.89. HR-MS of (*R*)-CNB in CH_3CN: Calculated for $C_{64}H_{64}N_2O_8$ $[M+H]^+$ 989.4735; found 989.4743.

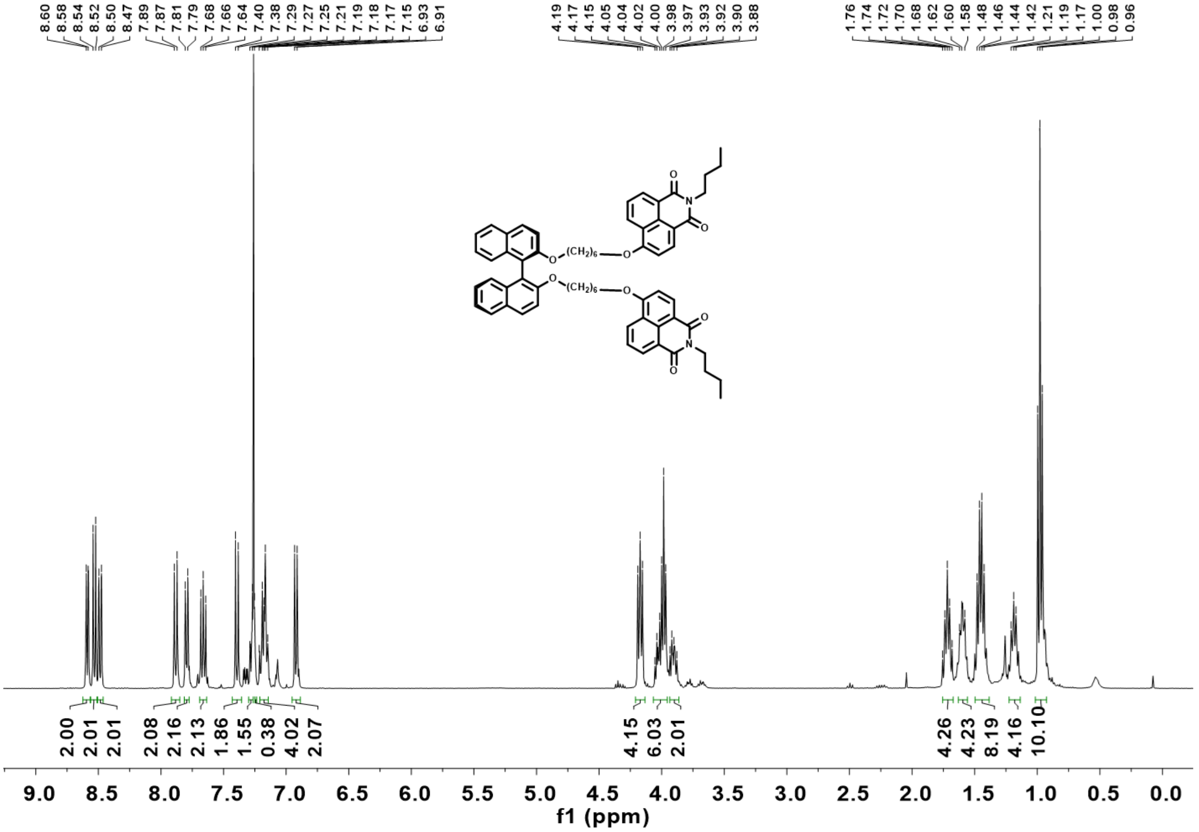

Supplementary Fig. 16 ^{1}H-NMR of (R)-CNB (400 MHz, $CDCl_3$).

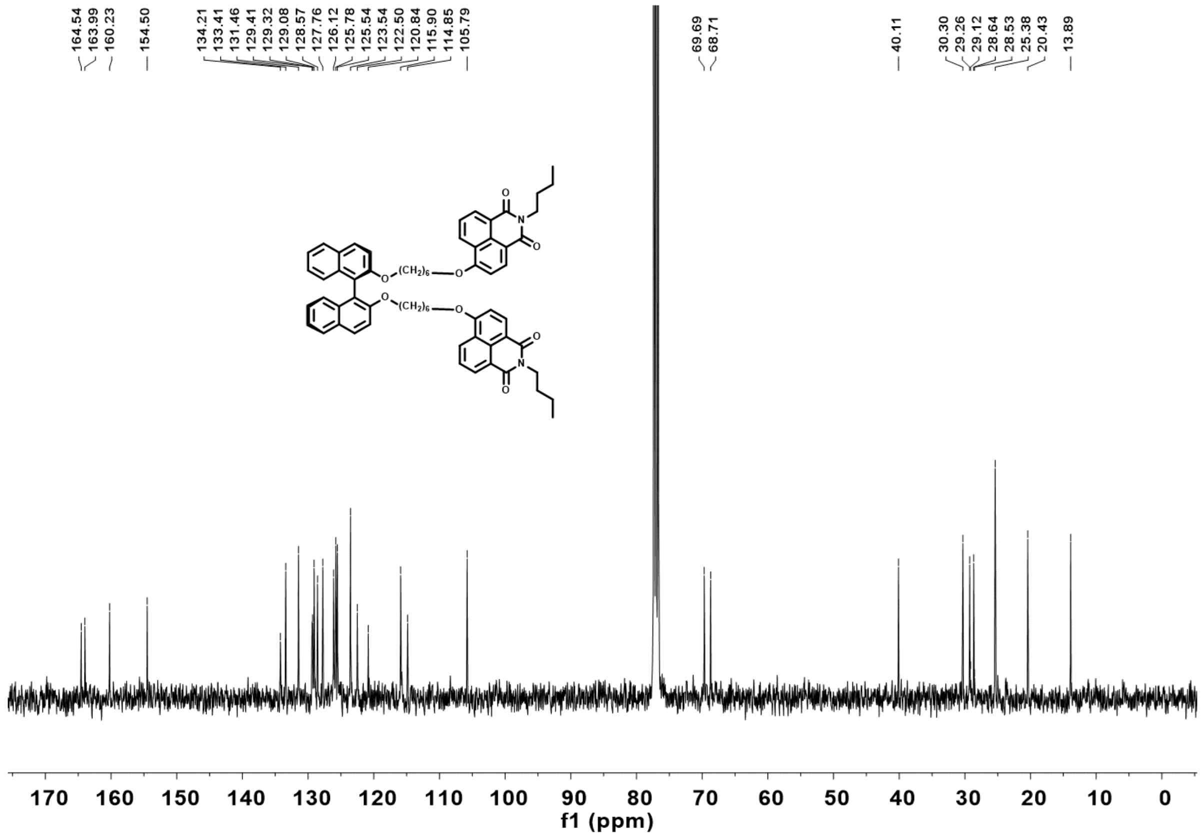

Supplementary Fig. 17 ^{13}C-NMR of (R)-CNB (101 MHz, $CDCl_3$).

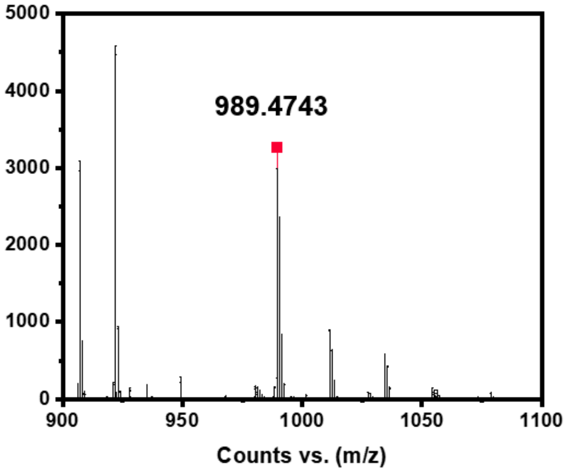

Supplementary Fig. 18 HR-MS of (R)-CNB in CH_3CN: Calculated for $C_{64}H_{64}N_2O_8$ $[M+H]^+$ 989.4735; found 989.4743.

2. Photophysical behaviors and property characterization

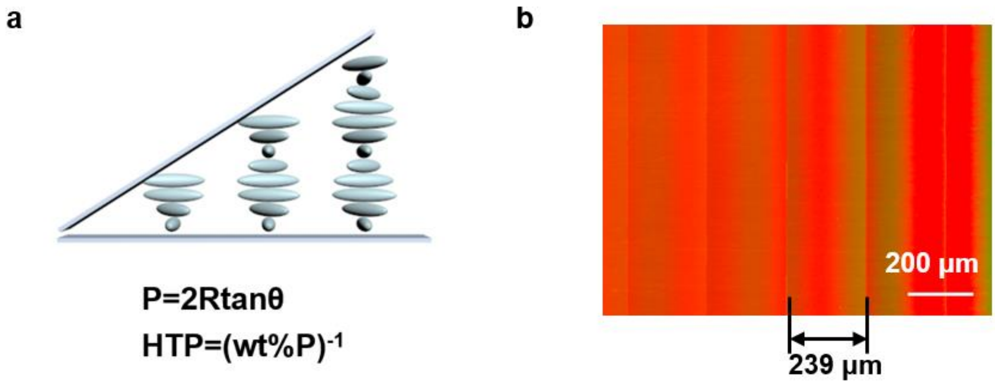

Supplementary Fig. 19 a Scheme illustration of HTP measurement using the Cano wedge method. **b** Cano lines of (S)-CNB in 5CB (1.0 wt%) observed by using POM.

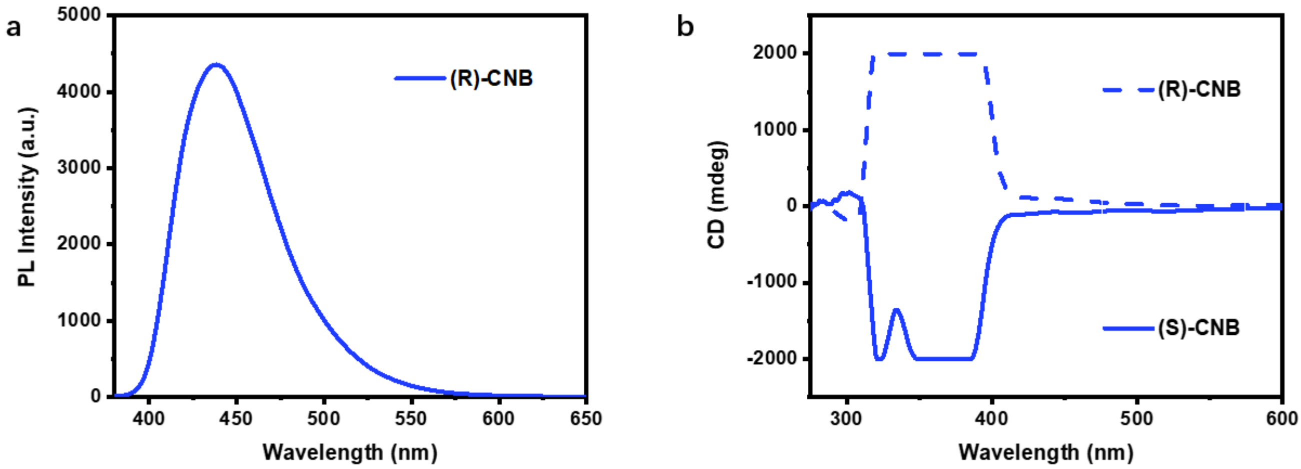

Supplementary Fig. 20 a Fluorescence spectrum of (R)-CNB in 5CB host (3.0 wt%, λ_{ex}=365 nm). **b** CD spectra of (S)-CNB and (R)-CNB in 5CB host (3.0 wt%).

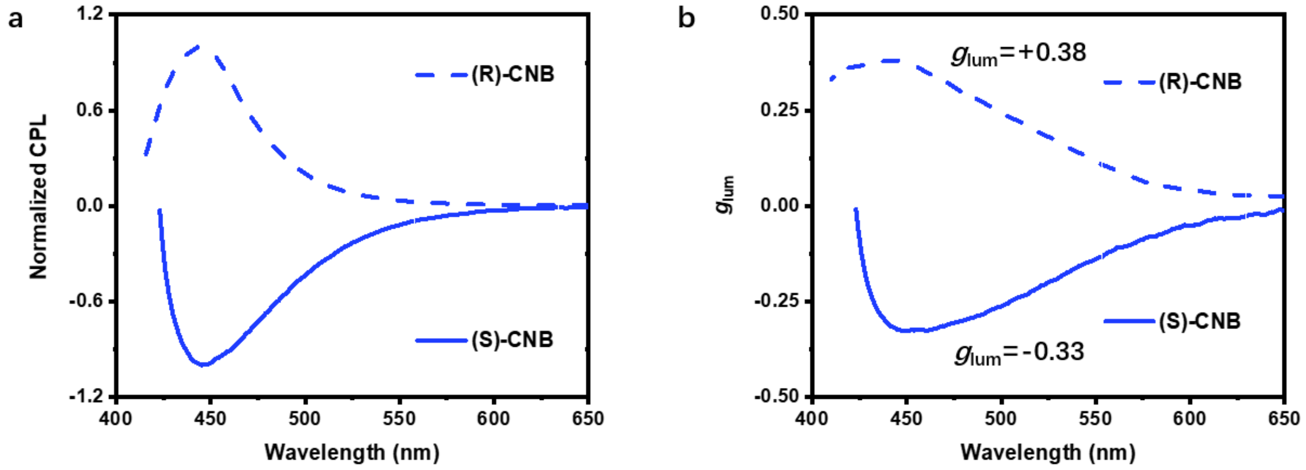

Supplementary Fig. 21 a Normalized CPL spectra of (R)-CNB and (S)-CNB in 5CB host (λ_{ex}=365 nm, 3.0 wt%). **b** g_{lum} spectra of (R)-CNB and (S)-CNB in 5CB host (λ_{ex}=365 nm, 3.0 wt%).

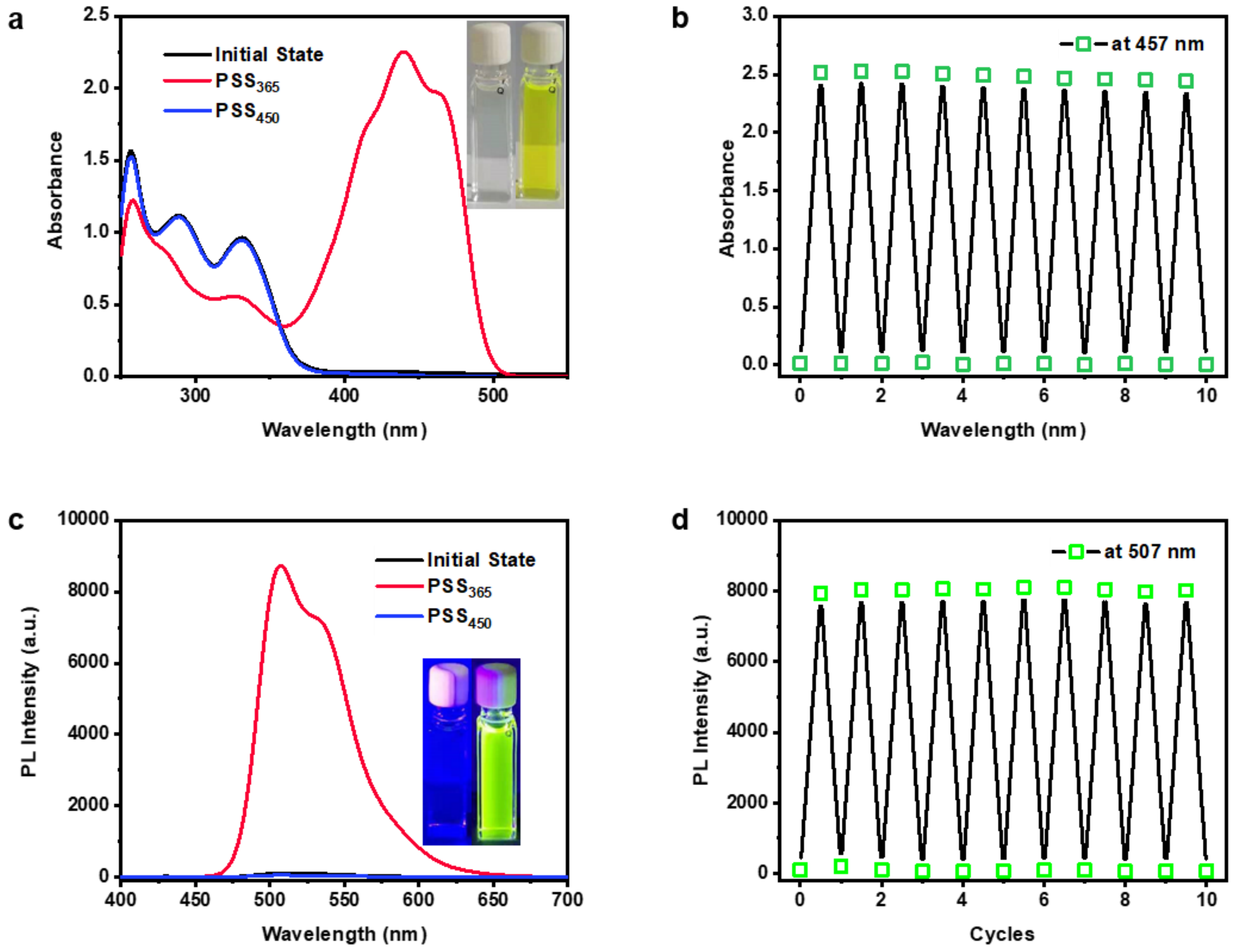

Supplementary Fig. 22 a Absorption spectra of DG in 1,4-dioxane solution at different states (10^{-5} M). **b**

Fatigue resistance of the absorption intensity at 457 nm of DG in dilute solution by alternating light irradiation with 365 and 450 nm. **c** Fluorescence spectra of DG in 1,4-dioxane solution at different states (λ_{ex}=365 nm, 10^{-5} M). **d** Fatigue resistance of the fluorescence intensity at 507 nm of DG in dilute solution by alternating light irradiation with 365 and 450 nm (λ_{ex}=365 nm, 10^{-5} M).

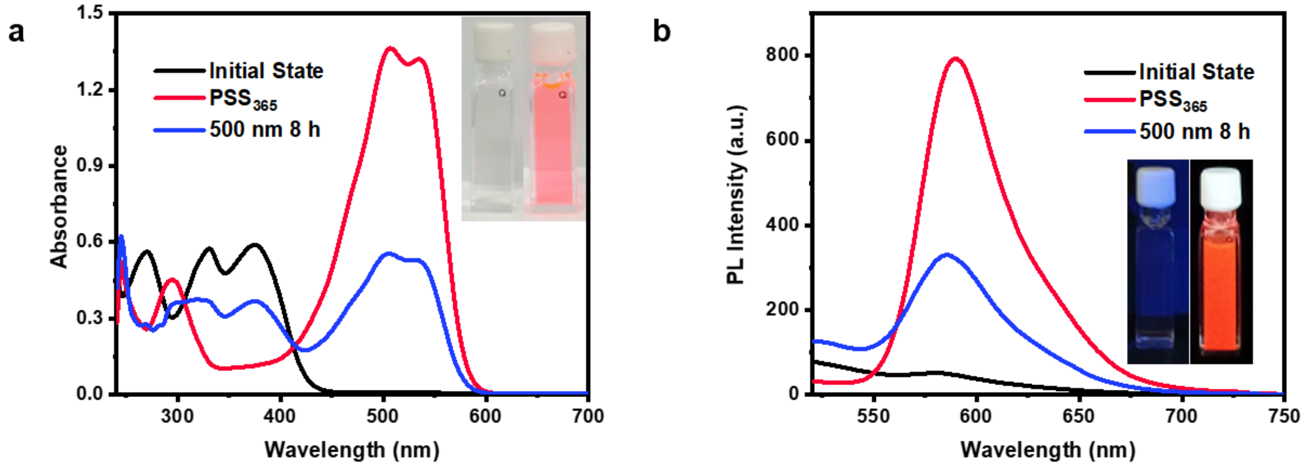

Supplementary Fig. 23 **a** Absorption spectra of DR in 1,4-dioxane solution at different states (10^{-5} M). **b** Fluorescence spectra of DR in 1,4-dioxane dilute solution at different states (λ_{ex}=365 nm, 10^{-5} M).

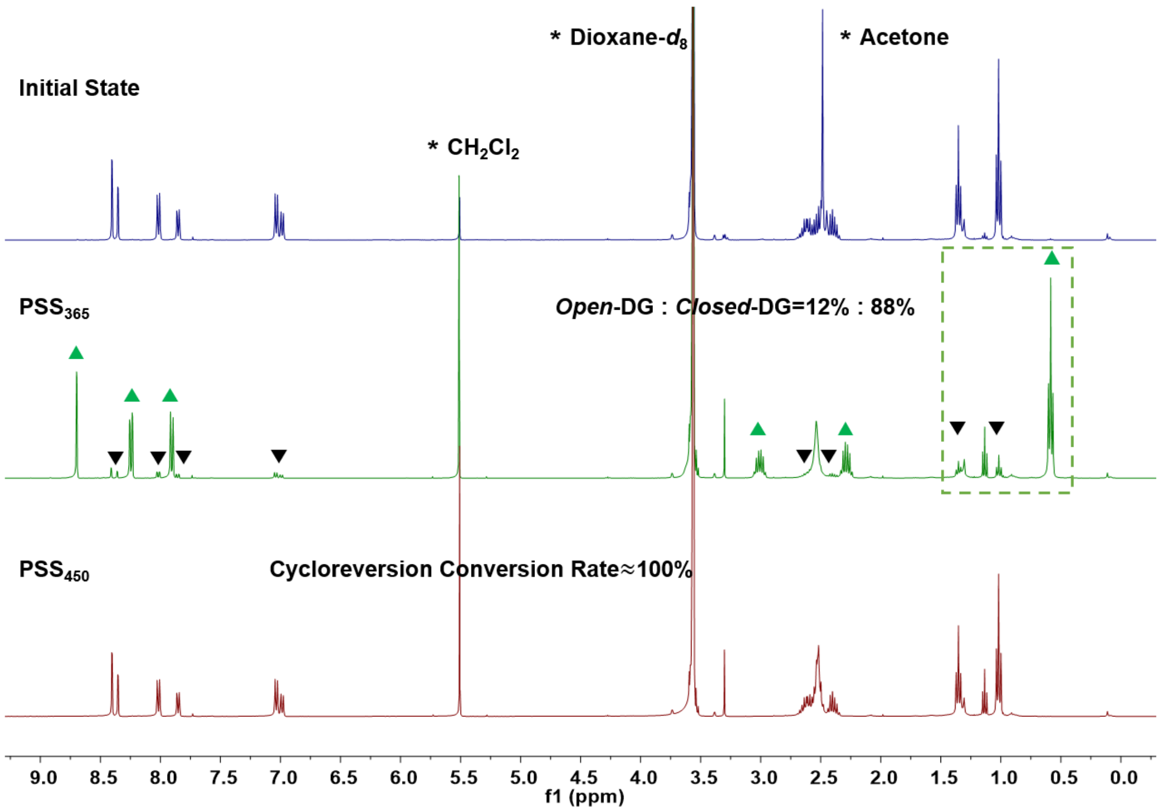

Supplementary Fig. 24 Photocyclization/cycloreversion of DG upon light irradiation with 365 nm and 450 nm, tracked by ^{1}H-NMR (400 MHz, 1,4-Dioxane-d_8).

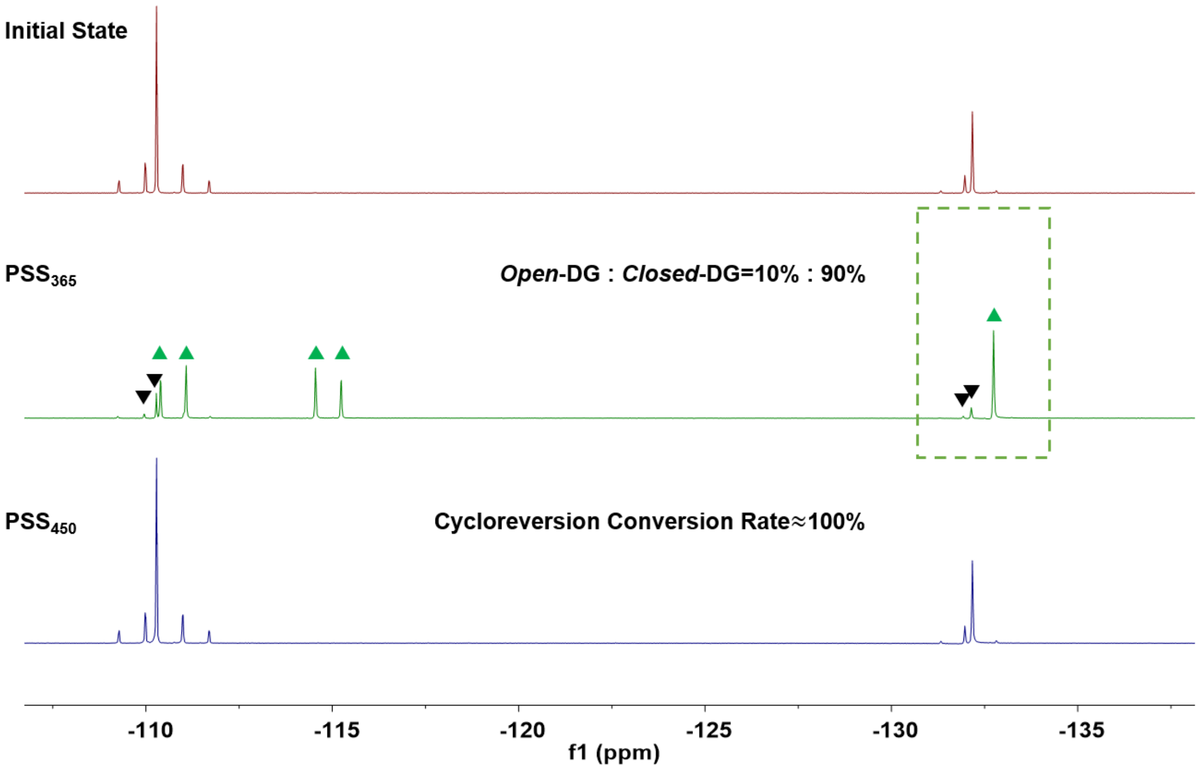

Supplementary Fig. 25 Photocyclization/cycloreversion of DG upon light irradiation with 365 nm and 450 nm, tracked by ^{19}F-NMR (377 MHz, 1,4-Dioxane-d_8).

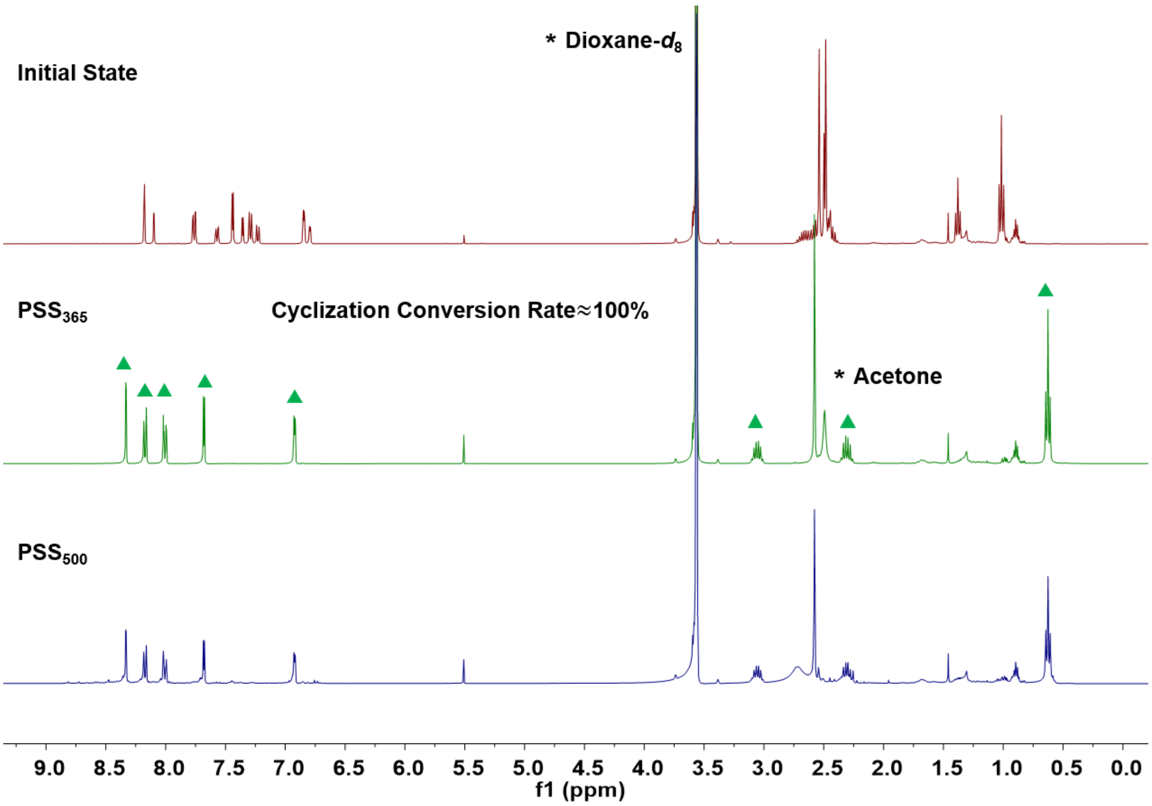

Supplementary Fig. 26 Photocyclization/cycloreversion of DR upon light irradiation with 365 nm and 500 nm, tracked by ^{1}H-NMR (400 MHz, 1,4-Dioxane-d_8).

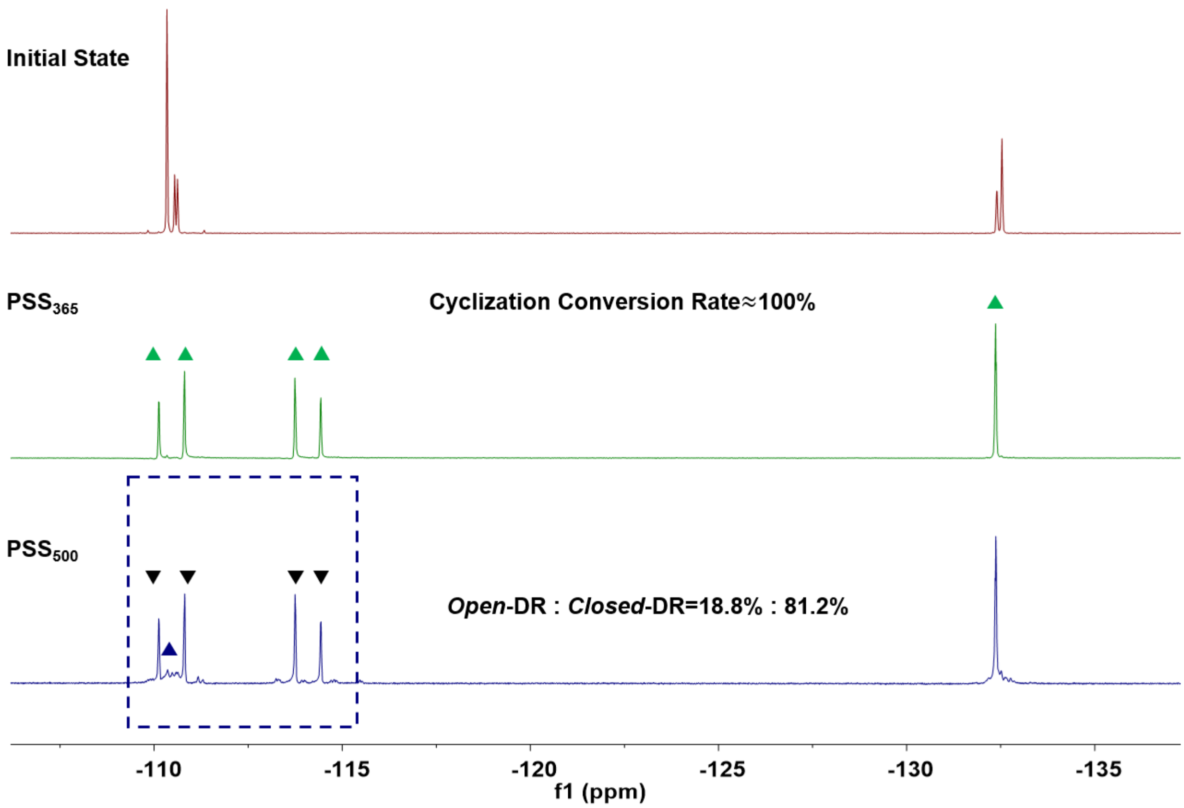

Supplementary Fig. 27 Photocyclization/cycloreversion of DR upon light irradiation with 365 nm and 500 nm, tracked by ^{19}F-NMR (377 MHz, 1,4-Dioxane-*d*$_8$).

3. Phototunable FRET systems

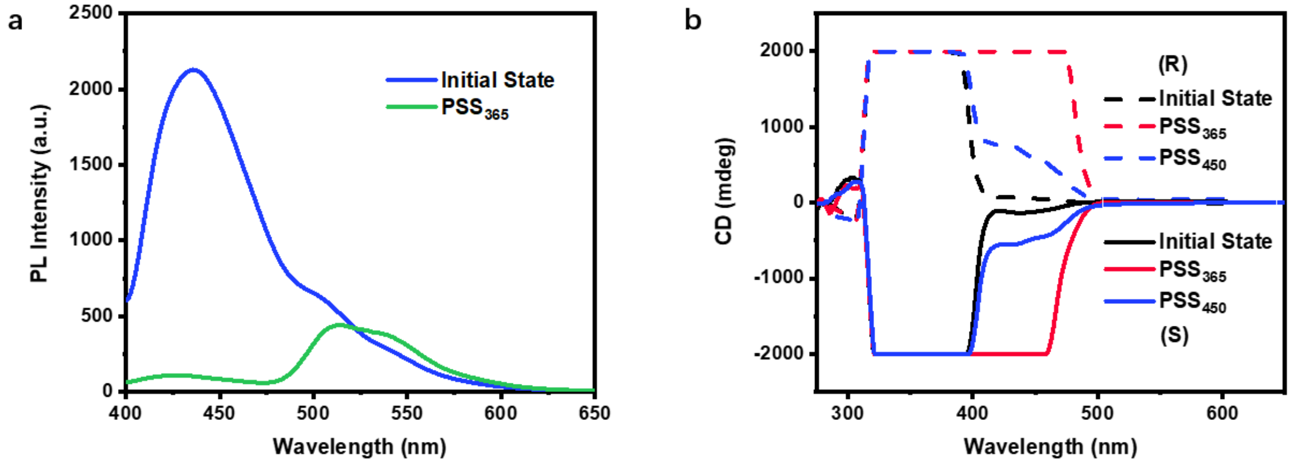

Supplementary Fig. 28 a Fluorescence spectra of (R)-LC-D ((R)-CNB:DG=3.0 wt%:1.5 wt%, λ_{ex}=365 nm) before and after UV irradiation (1.0 mW/cm^2). **b** CD spectra of (S)-LC-D and (R)-LC-D at different states.

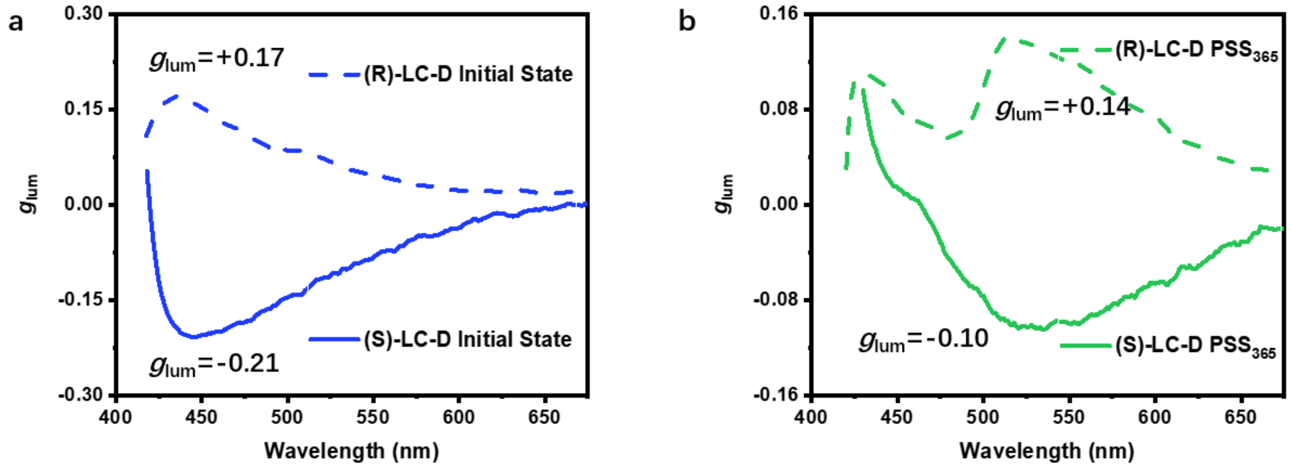

Supplementary Fig. 29 g_{lum} spectra of (S)-LC-D and (R)-LC-D systems **a** before and **b** after UV irradiation (CNB:DG=3.0 wt%:1.5 wt%, λ_{ex}=365 nm).

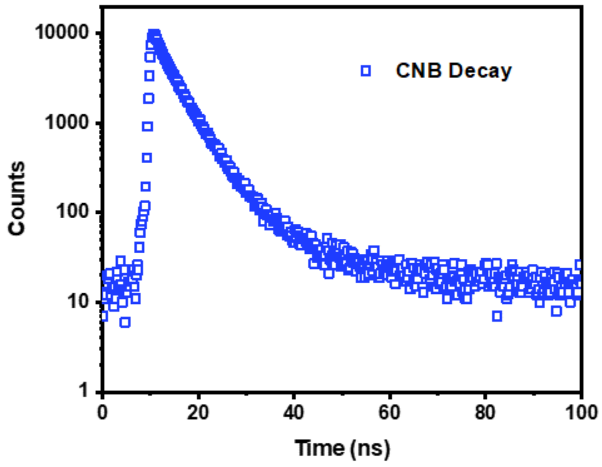

Supplementary Fig. 30 Fluorescence decay profile of (S)-CNB (3.0 wt% in 5CB) without the present of DG (λ_{ex}=365 nm, monitored at 436 nm).

Supplementary Table. 1 Fluorescence decay parameters (λ_{ex}=365 nm, $\lambda_{monitor}$=436 nm) of (S)-CNB in 5CB (3.0 wt%) host and (S)-LC-D system.

		τ_{av} (ns)	E	K_{ET}
3.0 wt% (S)-CNB in 5CB		4.601	/	/
(S)-LC-D	Initial State	3.308	28.1%	0.085
	PSS_{365}	0.860	81.3%	0.945
	PSS_{450}	3.195	30.6%	0.096

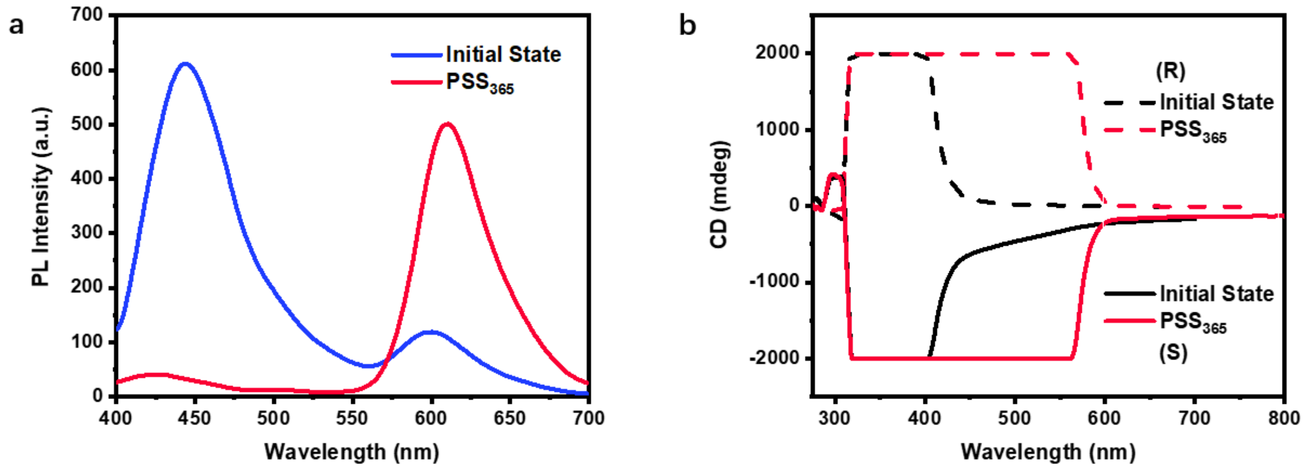

Supplementary Fig. 31 a Fluorescence spectra of (R)-LC-T ((R)-CNB:DG:DR=3.0 wt%:1.5 wt%:1.0 wt%, λ_{ex}=365 nm) before and after UV irradiation (1.0 mW/cm^2). **b** CD spectra of (S)-LC-T and (R)-LC-T at different states.

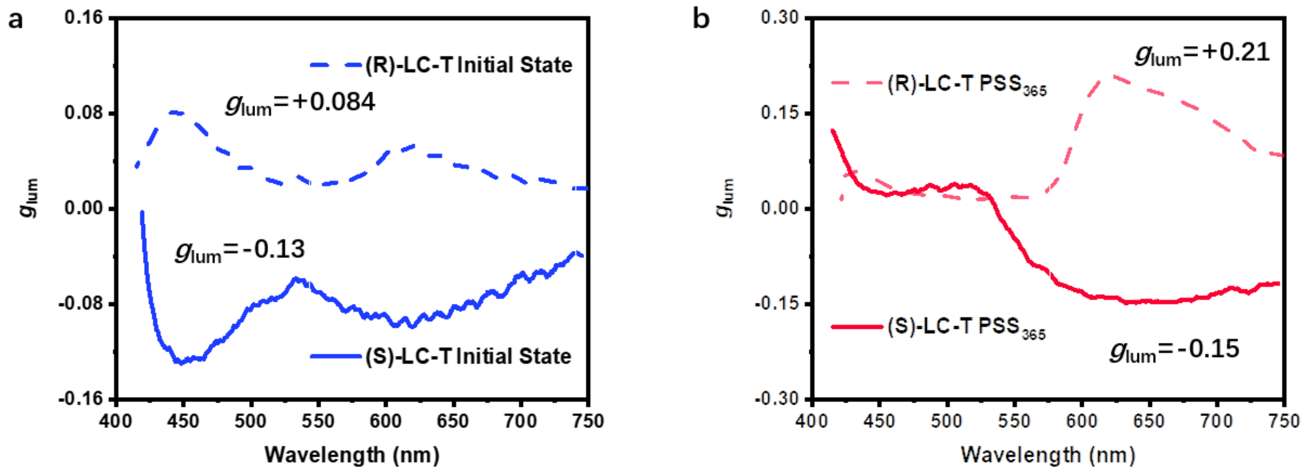

Supplementary Fig. 32 g_{lum} spectra of (S)-LC-T and (R)-LC-T systems **a** before and **b** after UV irradiation (CNB:DG:DR=3.0 wt%:1.5 wt%:1.0 wt%, λ_{ex}=365 nm).

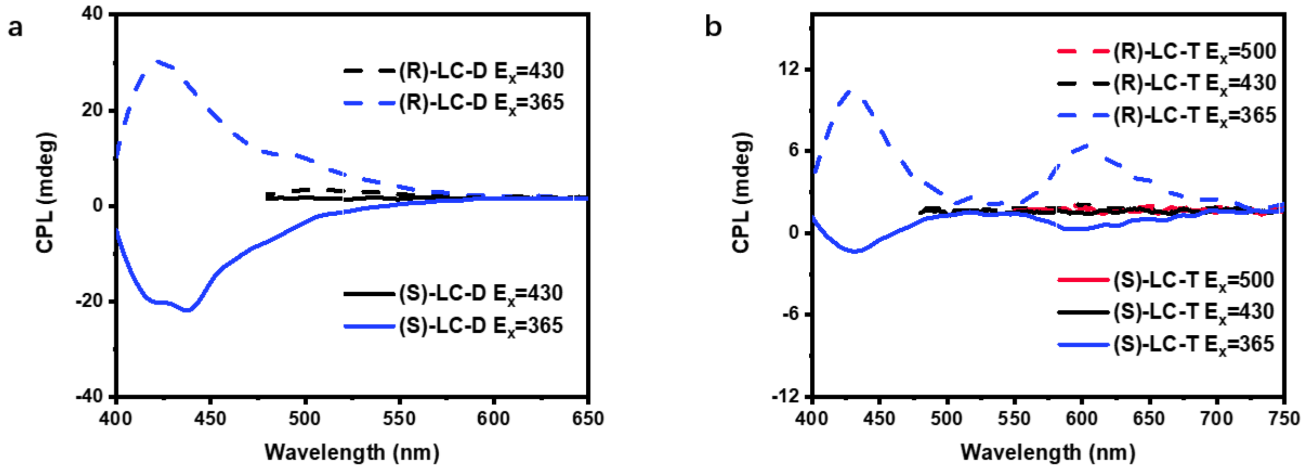

Supplementary Fig. 33 CPL spectra of **a** LC-D and **b** LC-T systems at initial state by excited light with different wavelength.

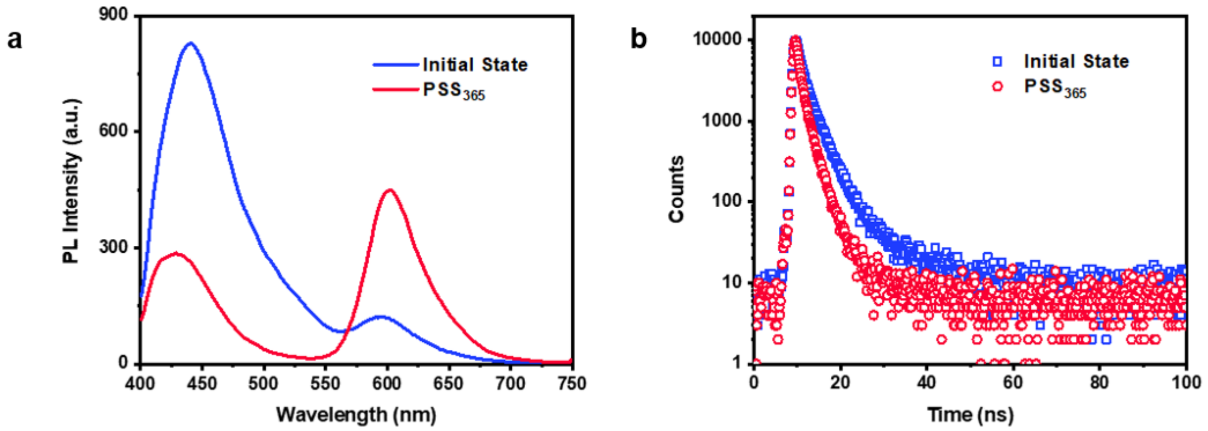

Supplementary Fig. 34 a Fluorescence spectra of (S)-LC-B/R system in different states (λ_{ex}=365 nm). **b** Fluorescence decay profile of (S)-CNB in (S)-LC-B/R system (λ_{ex}=365 nm, monitored at 434 nm).

Supplementary Table. 2 Fluorescence decay parameters (λ_{ex}=365 nm, $\lambda_{monitor}$=436 nm) of (*S*)-CNB in (*S*)-LC-T and (*S*)-LC-B/R system.

		τ_{av} (ns)	*E*	K_{ET}
(*S*)-LC-T	Initial State	3.183	30.8%	0.097
	PSS_{365}	0.278	94.0%	3.380
(*S*)-LC-B/R	Initial State	2.574	44.1%	0.171
	PSS_{365}	1.519	67.0%	0.441

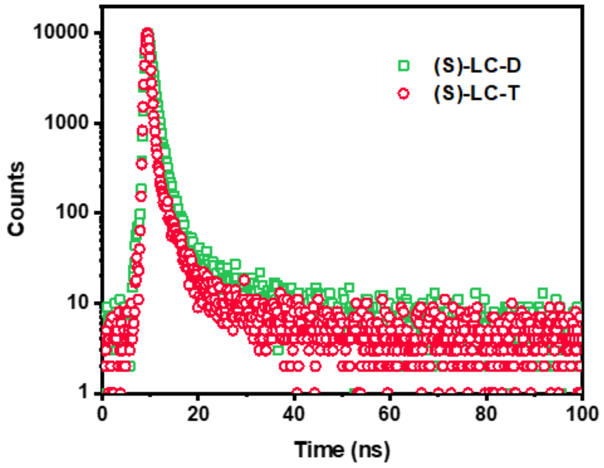

Supplementary Fig. 35 Fluorescence decay profile of DG with and without the present of DR (λ_{ex}=365 nm, monitored at 510 nm).

Supplementary Table. 3 Fluorescence decay parameters (λ_{ex}=365 nm, $\lambda_{monitor}$=510 nm) of DG in (S)-LC-D and (S)-LC-T system.

		τ_{av} (ns)	*E*	K_{ET}
(S)-LC-D	PSS_{365}	1.229	/	/
(S)-LC-T	PSS_{365}	0.584	52.5%	0.900

4. Characterization of Tricolor LCPCs

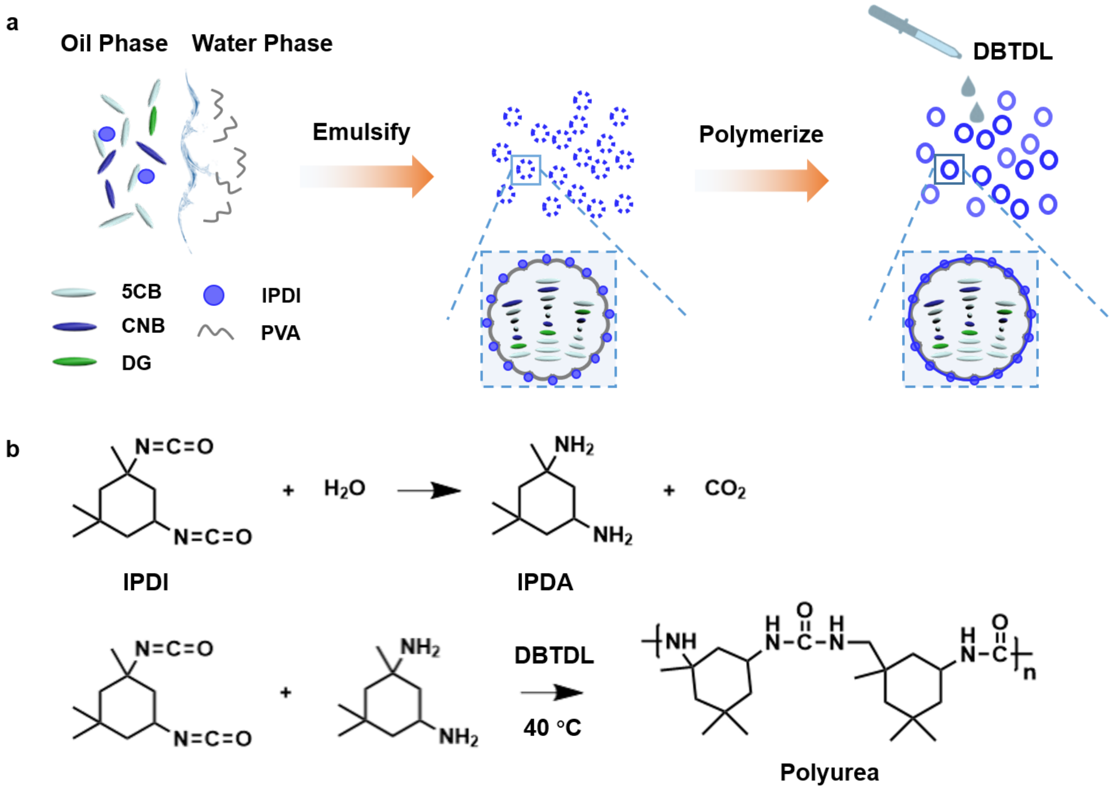

Supplementary Fig. 36 a Scheme illustration of the LCPC preparation through the interfacial polymerization. **b** Reaction process of the polyurea shell.

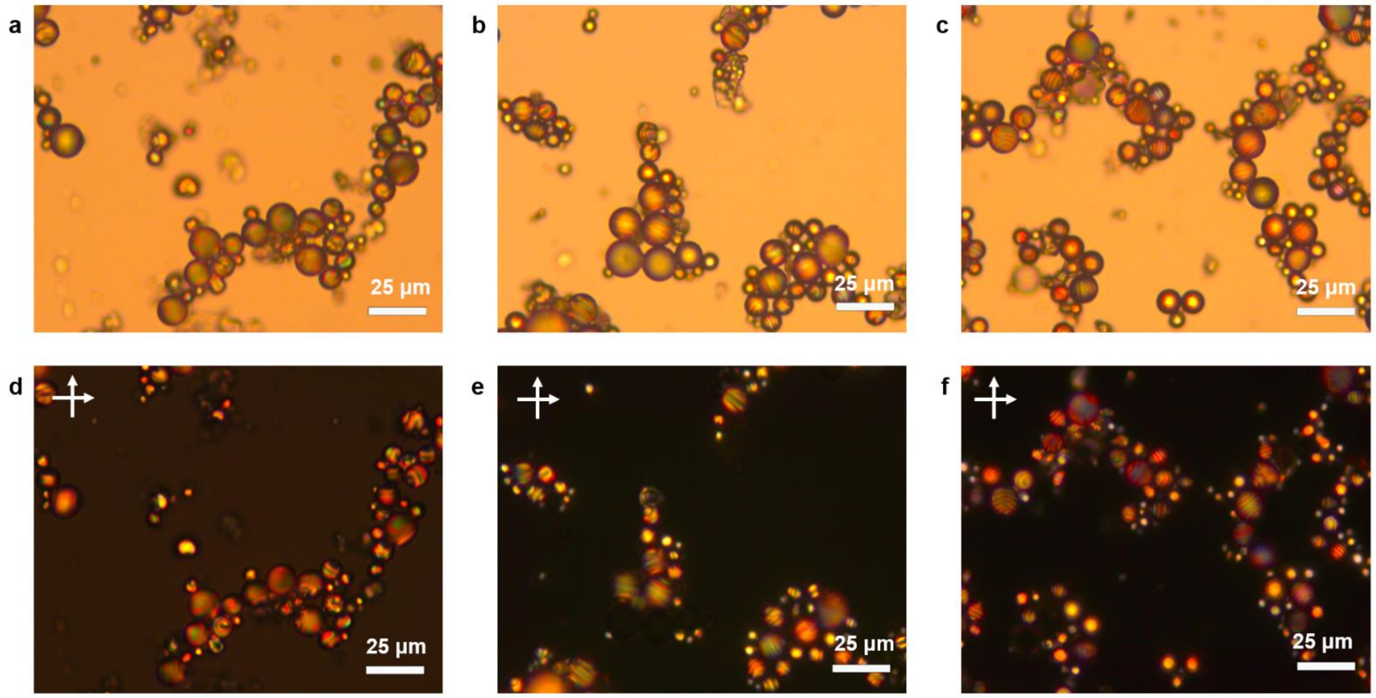

Supplementary Fig. 37 Optical microscope images of **a** LCPC-S; **b** LCPC-D; **c** LCPC-T. POM images under crossed polarizers of **d** LCPC-S; **e** LCPC-D; **f** LCPC-T.

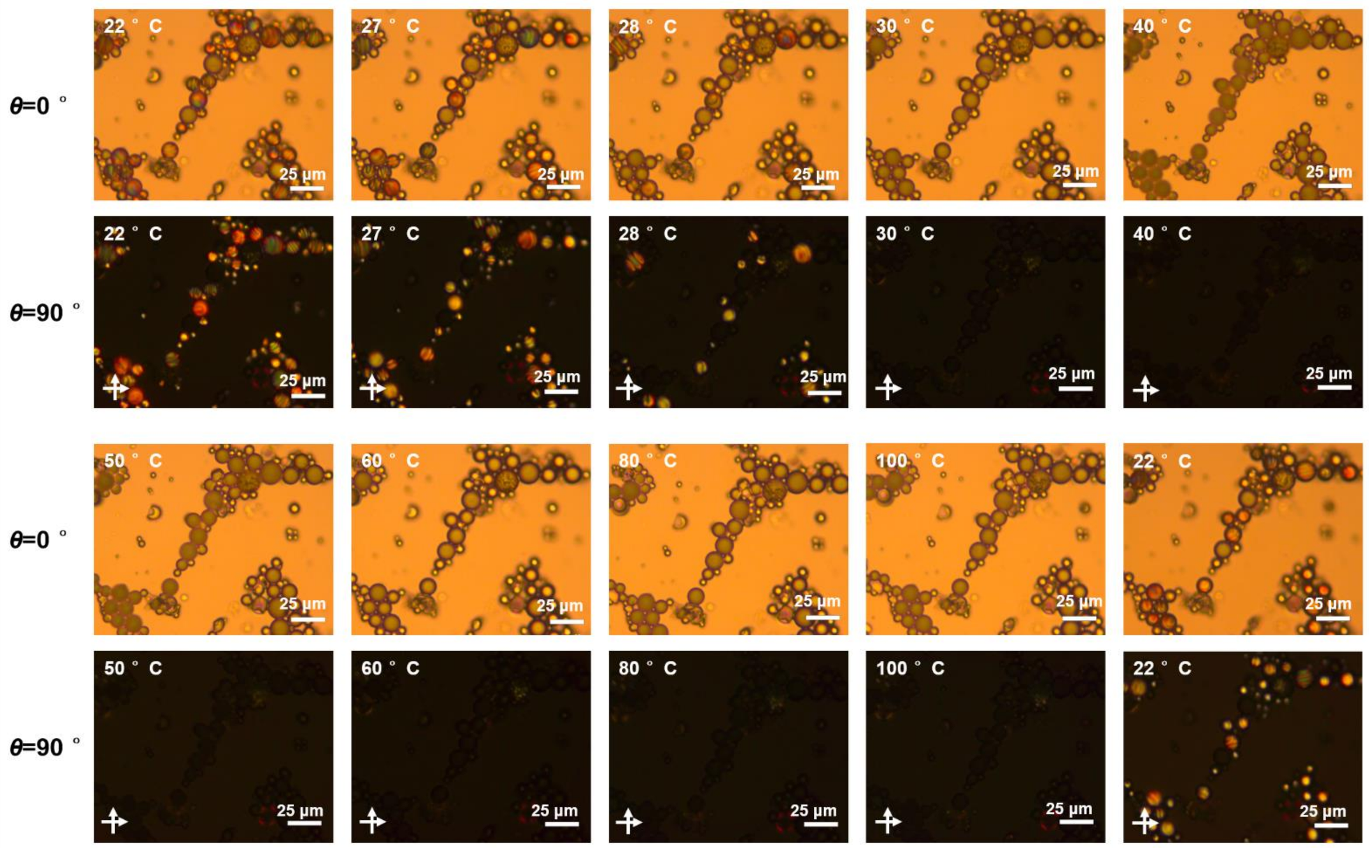

Supplementary Fig. 38 POM images of LCPC-D during the heating-cooling process.

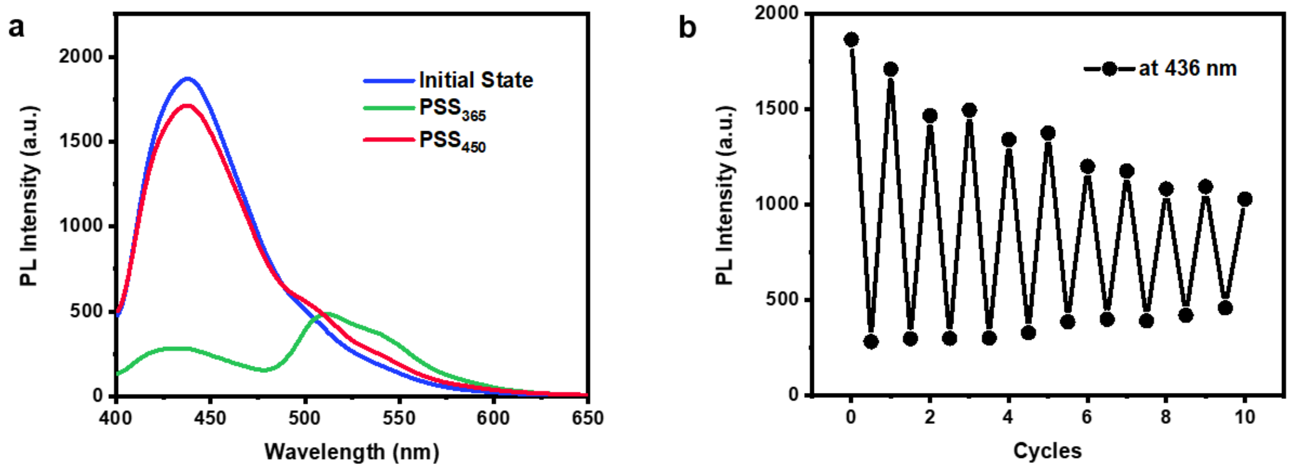

Supplementary Fig. 39 **a** Fluorescence spectra of LCPC-D in different states (λ_{ex}=365 nm). **b** Fatigue

resistance of the fluorescence intensity at 436 nm of LCPC-D by alternating light irradiation with 365 and 450 nm (λ_{ex}= 365 nm).

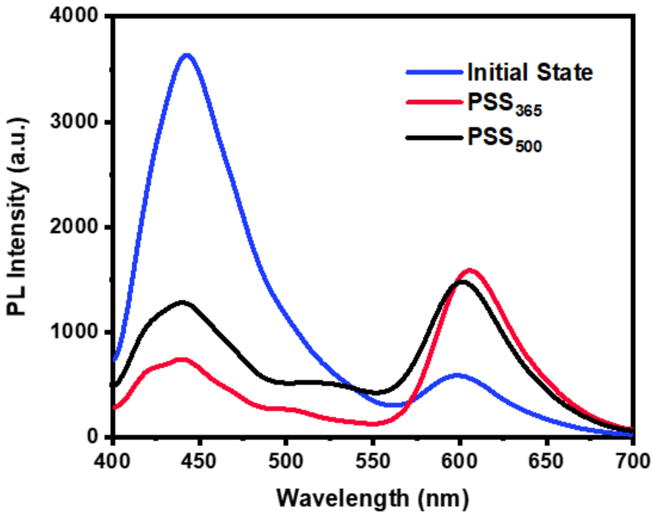

Supplementary Fig. 40 Fluorescence spectra of LCPC-T in different states (λ_{ex}=365 nm).

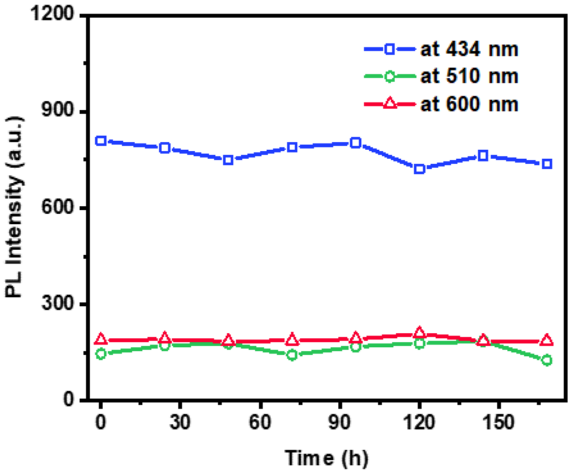

Supplementary Fig. 41 Fluorescence intensity at 434 nm of LCPC-S, 510 nm of LCPC-D, and 600 nm of LCPC-T when stored at room temperature for one week.

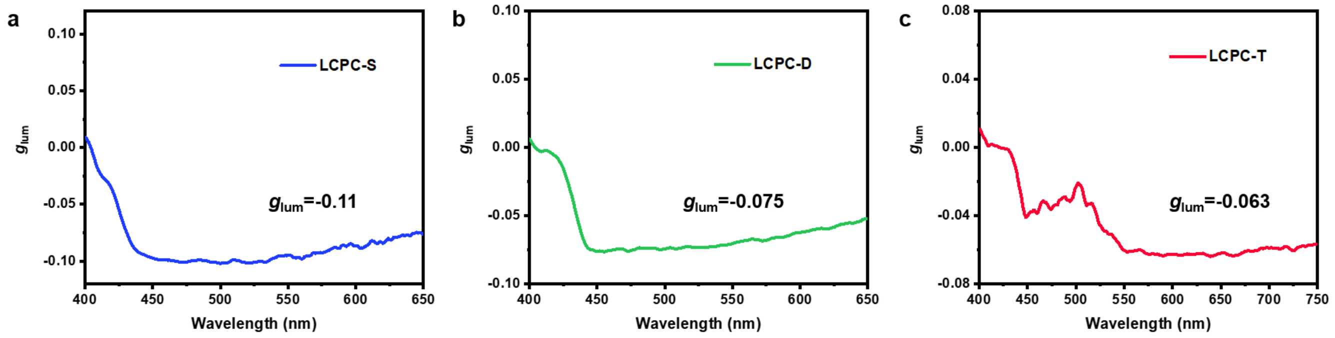

Supplementary Fig. 42 g_{lum} spectra of **a** LCPC-S; **b** LCPC-D; **c** LCPC-T (λ_{ex}=365 nm).

5. Phototunable Full-color CPL and Fluorescence

Supplementary Table. 4 Composition of tricolor LCPCs for different colored films

Film color	Amount of LC Microcapsules (mg)			Mass Ratio
	LCPC-S	LCPC-D	LCPC-T	
Light Blue	9	3	/	3 : 1
Cyan	4	10	/	1 : 2.5
Purple	9	/	3	3 : 1
Pink	5	/	5	1 : 1
Yellow	/	8	2	4 : 1
Orange	/	5	5	1 : 1
White	4	6	1	4: 6 : 1

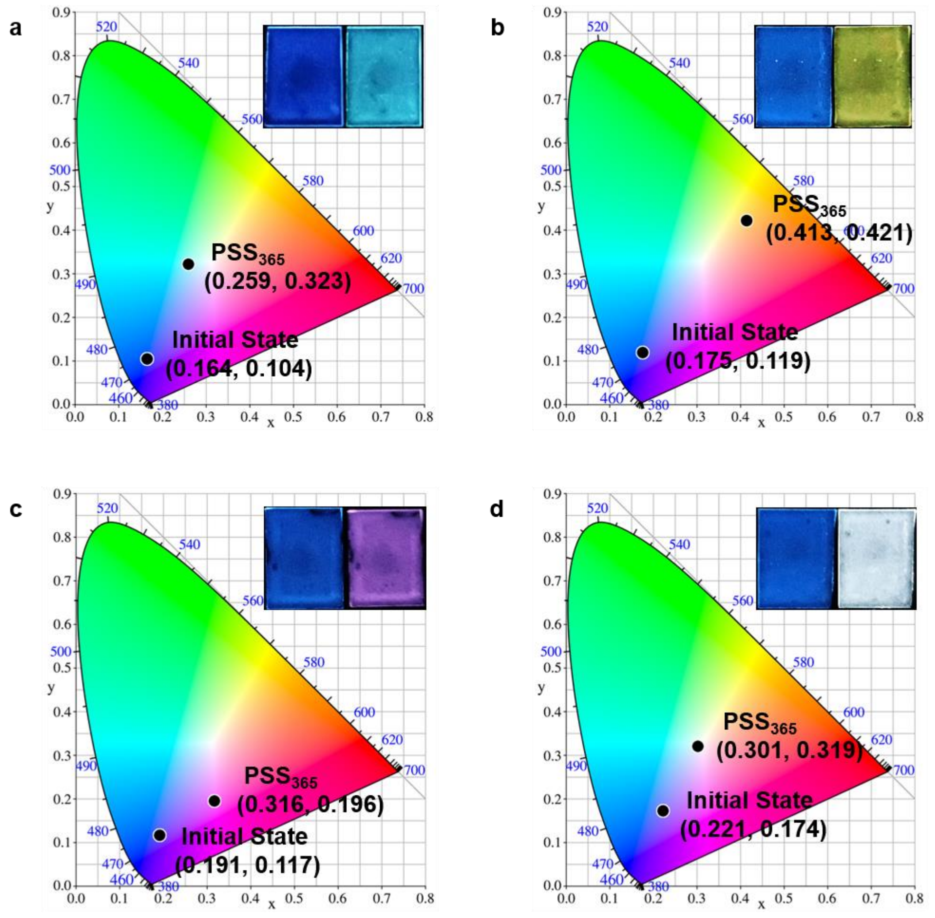

Supplementary Fig. 43 a CIE chromaticity diagram of S/D in different states, inset images are the fluorescent photographs. **b** CIE chromaticity diagram of D/T in different states, inset images are the fluorescent photographs. **c** CIE chromaticity diagram of S/T in different states, inset images are the fluorescent photographs. **d** CIE chromaticity diagram of S/D/T in different states, inset images are the fluorescent photographs.

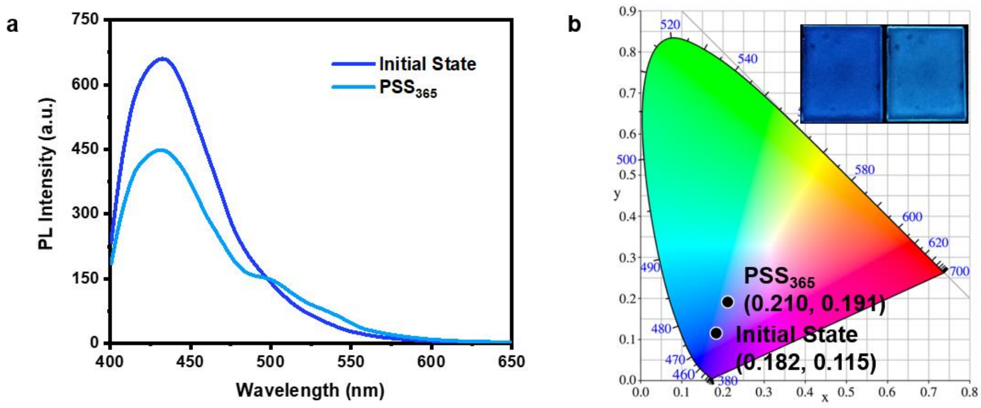

Supplementary Fig. 44 a Fluorescence spectra of LCPC film with light blue color in different states. **b** CIE chromaticity diagram of LCPC film with light blue color in different states, inset images are the fluorescent photographs.

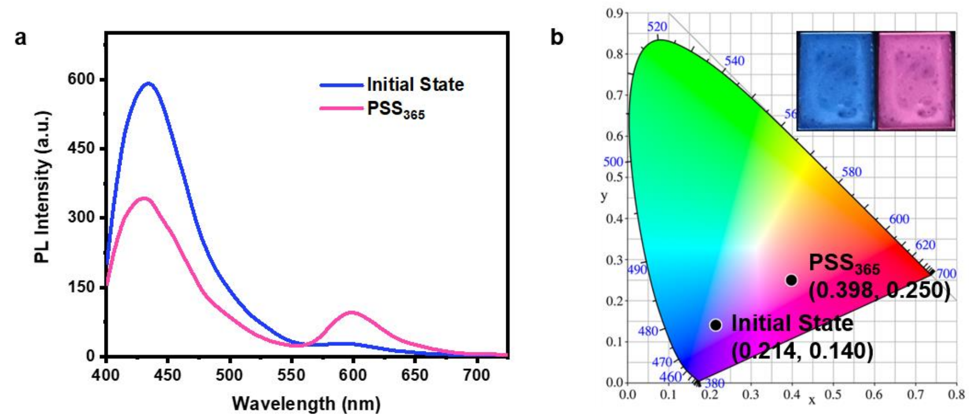

Supplementary Fig. 45 a Fluorescence spectra of LCPC film with pink color in different states. **b** CIE chromaticity diagram of LCPC film with pink color in different states, inset images are the fluorescent

photographs.

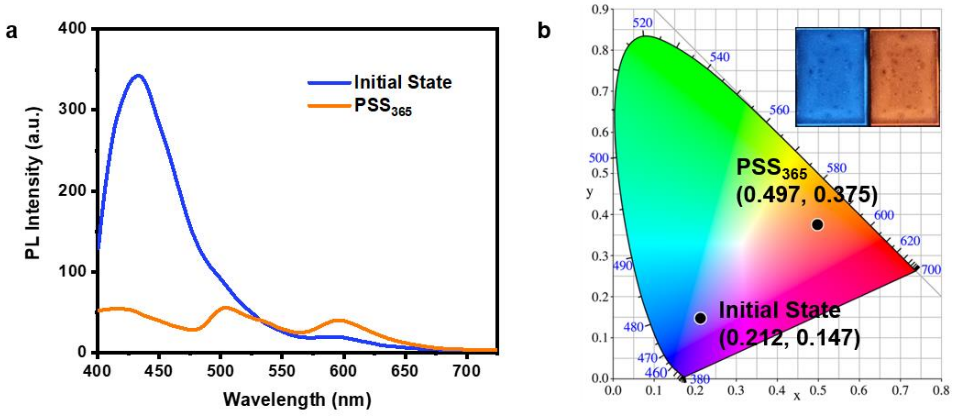

Supplementary Fig. 46 a Fluorescence spectra of LCPC film with orange color in different states. **b** CIE chromaticity diagram of LCPC film with orange color in different states, inset images are the fluorescent photographs.

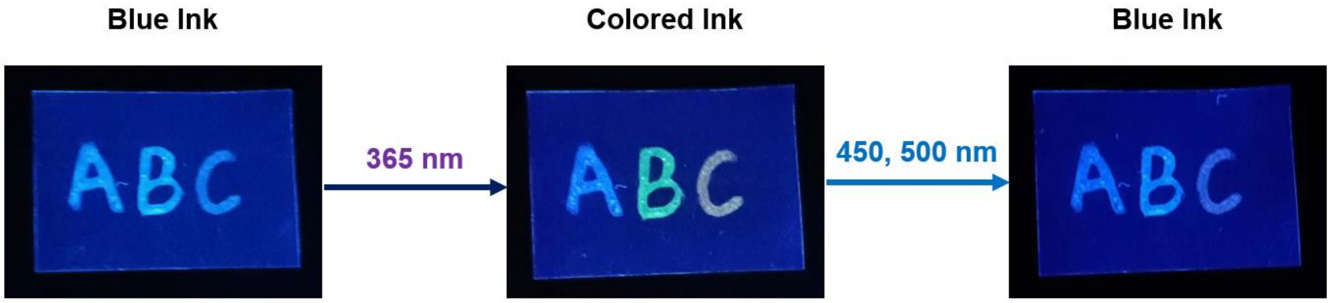

Supplementary Fig. 47 Fluorescent images of colored letters by direct ink writing at different states.

6. Information encryption

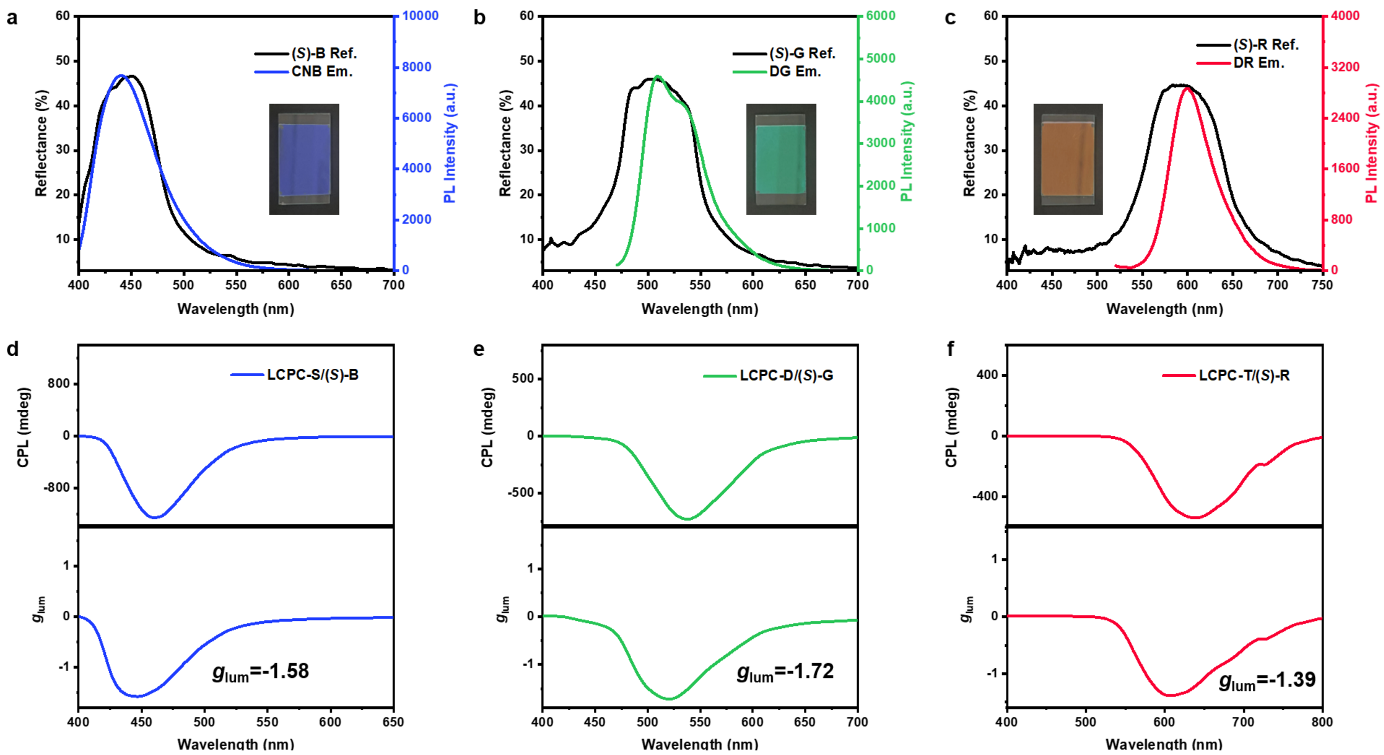

Supplementary Fig. 48 a Fluorescence spectrum of (S)-CNB and reflection spectrum of (S)-B film. **b** Fluorescence spectrum of DG at PSS_{365} and reflection spectrum of (S)-G film. **c** Fluorescence spectrum of DR at PSS_{365} and reflection spectrum of (S)-R film. CPL and g_{lum} spectra of **d** LCPC-S/(S)-B, **e** LCPC-D/(S)-G, **f** LCPC-T/(S)-R.

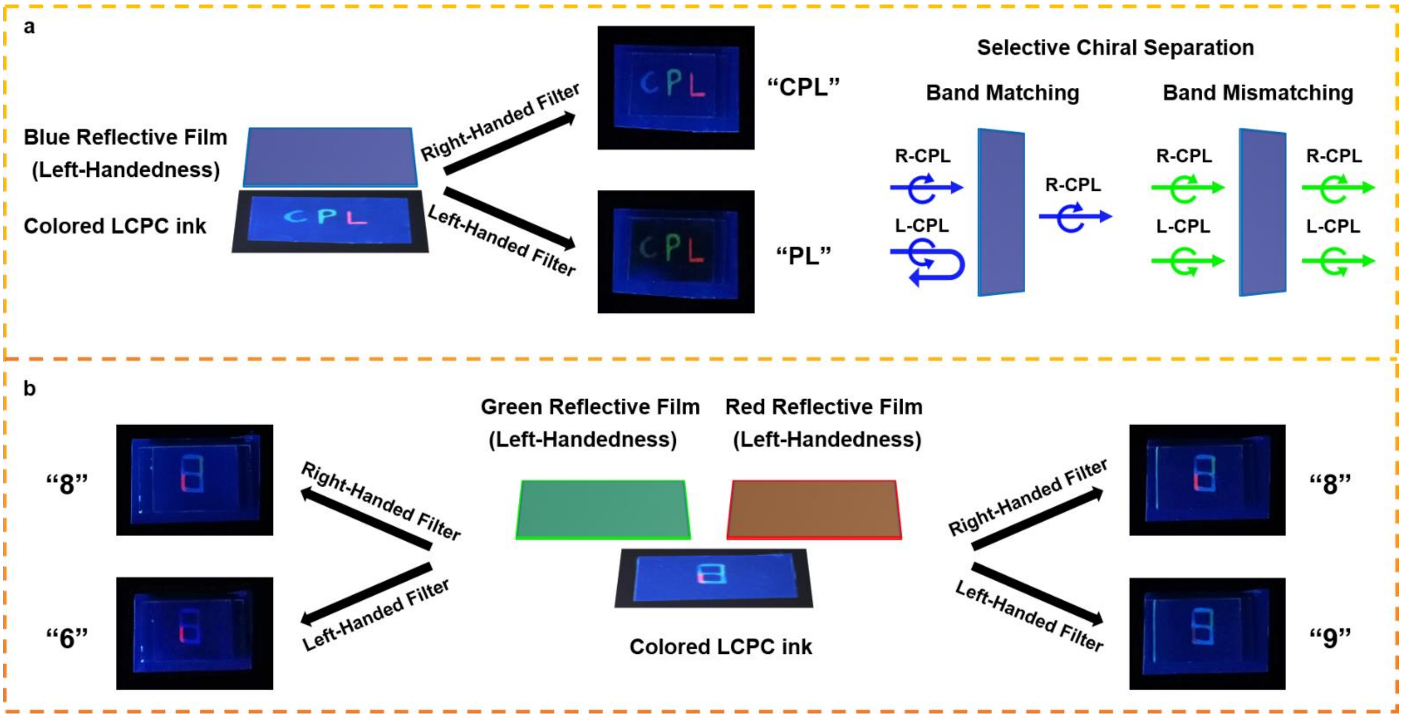

Supplementary Fig. 49 a Scheme illustration of selective chiral separation of CLC reflective films and the CPL images of double-layer device under left-polarized and right-polarized windows. **b** Visualized CPL information decryption using CLC reflective films with different photonic band gaps as the decipher.

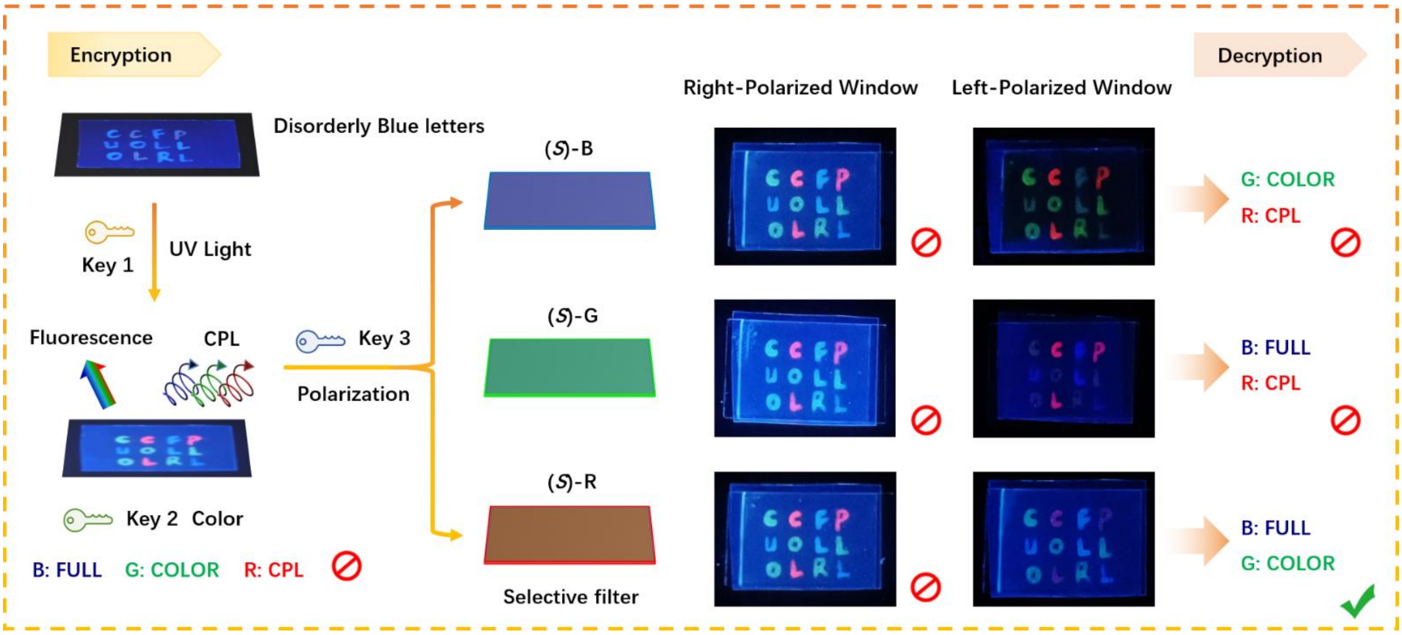

Supplementary Fig. 50 Demonstration of a letter array for visualized 3D information decryption.

7. Calculation of energy levels

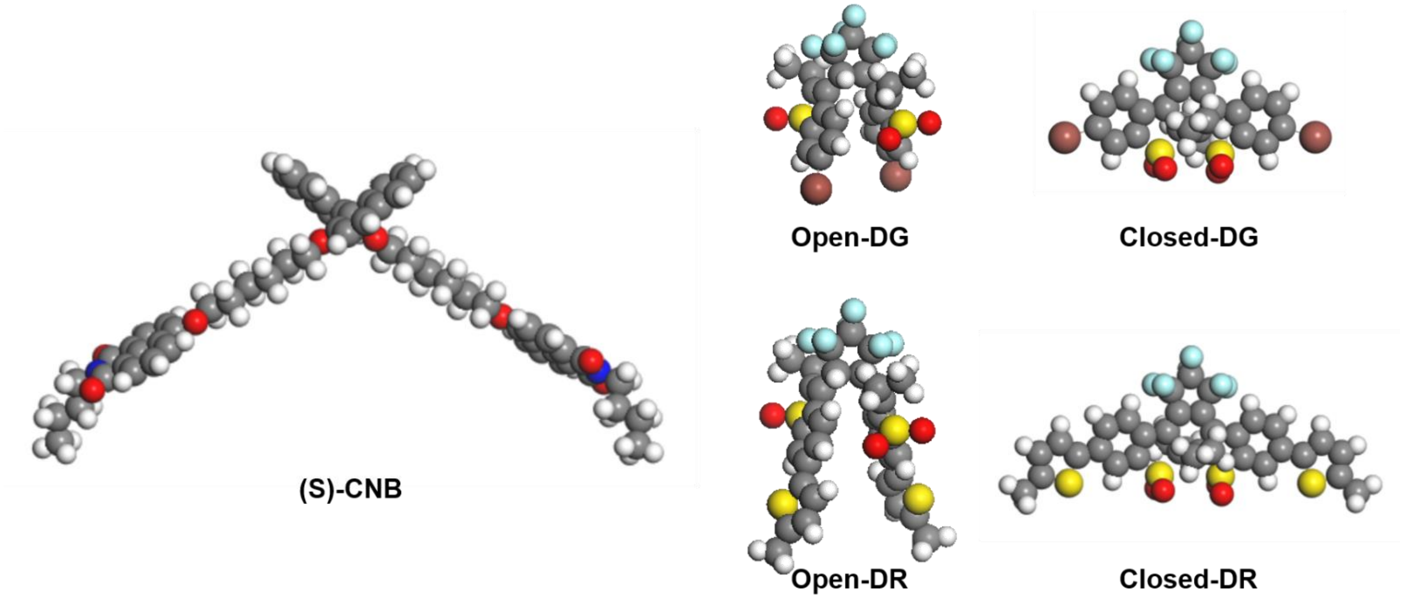

Supplementary Fig. 51 Optimized structures of (S)-CNB, DG, and DR calculated by Gaussian 09.

Supplementary Table. 5 Singlet transitions and corresponding energy gaps, absorption wavelengths, and oscillator strengths of (S)-CNB, obtained by TD-DFT calculations.

	Excitation	Energy (eV)	Wavelength (nm)	Oscillator Strength (f)
**(S)-CNB**	S1 HOMO→LUMO (99.9%)	3.0230	410.13	0.0000
	S5 HOMO-2→LUMO (43.4%)	3.5590	348.37	0.4273
	S6 HOMO-3→LUMO+1 (43.3%)	3.5625	348.02	0.1388

Supplementary Table. 6 Singlet transitions and corresponding energy gaps, absorption wavelengths, and oscillator strengths of the two isomers of DG, obtained by TD-DFT calculations.

Isomer	Excitation	Energy (eV)	Wavelength (nm)	Oscillator Strength (f)
**open-DG**	S1 HOMO→LUMO (96.9%)	3.5690	347.39	0.0414
	S2 HOMO-1→LUMO (93.8%)	3.6229	342.23	0.0835
	S3 HOMO→LUMO+1 (93.3%)	3.6642	338.37	0.0741
**closed-DG**	S1 HOMO→LUMO	2.4954	496.85	0.6510

	(98.0%)			
	S3 HOMO-2→LUMO (95.8%)	2.8456	435.71	0.0266
	S4 HOMO-4→LUMO (85.6%)	3.0003	413.24	0.0211

Supplementary Table. 7 Singlet transitions and corresponding energy gaps, absorption wavelengths, and oscillator strengths of the two isomers of DR, obtained by TD-DFT calculations.

Isomer	Excitation	Energy (eV)	Wavelength (nm)	Oscillator Strength (f)
**open-DR**	S1 HOMO→LUMO (95.3%)	2.8988	427.71	0.0229
	S3 HOMO→LUMO+1 (56.9%)	3.0972	400.30	0.1540
	S4 HOMO-1→LUMO+1 (46.8%)	3.1281	396.36	0.3479
**closed-DR**	S1 HOMO→LUMO (99.9%)	2.0874	593.95	1.2630
	S2 HOMO-1→LUMO (94.3%)	2.5308	489.91	0.0316
	S4 HOMO-3→LUMO (75.2%)	2.9528	419.89	0.0171

References

1. Tian, M., Wang, C., Ma, Q., Bai, Y., Sun, J. & Ding, C. A Highly Selective Fluorescent Probe for Hg^{2+} Based on a 1,8-Naphthalimide Derivative. *ACS Omega* **5**, 18176-18184 (2020).

2. Sheng, Y., Shen, D., Zhang, W., Zhang, H., Zhu, C. & Cheng, Y. Reversal Circularly Polarized Luminescence of AIE-Active Chiral Binaphthyl Molecules from Solution to Aggregation. *Chemistry* **21**, 13196-13200 (2015).

3. Lin, S., Zeng, S., Li, Z., Fan, Q. & Guo, J. Turn-On Mode Circularly Polarized Luminescence in Self-Organized Cholesteric Superstructure for Active Photonic Applications. *ACS Appl. Mater. Interfaces* **14**, 30362-30370 (2022).
